# Diversity of Pol IV Function Is Defined by Mutations at the Maize *rmr7* Locus

**DOI:** 10.1371/journal.pgen.1000706

**Published:** 2009-11-20

**Authors:** Jennifer L. Stonaker, Jana P. Lim, Karl F. Erhard, Jay B. Hollick

**Affiliations:** Department of Plant and Microbial Biology, University of California Berkeley, Berkeley, California, United States of America; The University of North Carolina at Chapel Hill, United States of America

## Abstract

Mutations affecting the heritable maintenance of epigenetic states in maize identify multiple small RNA biogenesis factors including NRPD1, the largest subunit of the presumed maize Pol IV holoenzyme. Here we show that mutations defining the *required to maintain repression7* locus identify a second RNA polymerase subunit related to *Arabidopsis* NRPD2a, the sole second largest subunit shared between *Arabidopsis* Pol IV and Pol V. A phylogenetic analysis shows that, in contrast to representative eudicots, grasses have retained duplicate loci capable of producing functional NRPD2-like proteins, which is indicative of increased RNA polymerase diversity in grasses relative to eudicots. Together with comparisons of *rmr7* mutant plant phenotypes and their effects on the maintenance of epigenetic states with parallel analyses of NRPD1 defects, our results imply that maize utilizes multiple functional NRPD2-like proteins. Despite the observation that RMR7/NRPD2, like NRPD1, is required for the accumulation of most siRNAs, our data indicate that different Pol IV isoforms play distinct roles in the maintenance of meiotically-heritable epigenetic information in the grasses.

## Author Summary

Multicellular plants possess a unique set of DNA–dependent RNA polymerase complexes (RNAPs) that prevent certain repetitious regions of the genome from being copied into stable RNAs. Two distinct RNAPs, termed Pol IV and Pol V, are required for this type of genome-silencing behavior in the eudicot *Arabidopsis thaliana*, but the mechanism by which these RNAPs accomplish this function is still relatively unknown. Using genetic and molecular methodologies, we identified a Pol IV–type subunit protein as being involved in a process of meiotically-heritable gene silencing in the maize plant known as paramutation. Our analyses of the available plant genome sequences indicate that monocots have a greater potential for RNAP diversity due to having duplicate variants of this particular subunit. Consistent with this inferred diversity, comparative analyses with plants defective in a different core Pol IV subunit indicate that the Pol IV–type RNAP in maize has distinct functional isoforms. The mechanistic and biological role(s) of these specific RNAPs in mediating genome regulation and heritable gene silencing in large genome cereals should now be tractable by biochemical approaches.

## Introduction

Plants have two DNA-dependent RNA polymerases, Pol IV and Pol V, in addition to the ubiquitous eukaryotic polymerases I, II, and III. Pol IV and Pol V arose specifically in land plants from an ancient duplication of the catalytic largest and second largest subunits of Pol II [1]. Subunits for these plant-specific RNA polymerases (RNAPs) were originally identified in the *Arabidopsis* genome [2] and subsequently in genetic screens for factors involved in small RNA-mediated transgene silencing [3],[4]. In *Arabidopsis*, mutations in the loci encoding the largest or shared second largest subunits of Pol IV and Pol V do not affect viability or development but do have distinct molecular effects on small RNA silencing pathways [5],[6]. Pol IV is required for the accumulation of 24 nt RNAs while Pol V produces non-coding RNA transcripts at low levels [5],[7],[8].

In maize, the largest subunit (NRPD1) of the presumed Pol IV functions with Required to Maintain Repression1 (RMR1), a Snf2-domain containing protein, and Mediator of Paramutation1 (MOP1), a putative RNA-dependent RNA polymerase related to *Arabidopsis* RDR2, to maintain meiotically-heritable epigenetic states at the *purple plant1* (*pl1*) and *colored plant1* (*b1*) loci [9]–[12]. The *pl1* and *b1* loci encode transcriptional activators of anthocyanin biosynthesis. Specific alleles of *pl1* and *b1*, namely *Pl1-Rhoades* and *B1-Intense*, exist in distinct epigenetic states characterized by different pigment levels. The *Pl-Rh* and *B-I* states are highly expressed and confer dark pigmentation to plant tissues while the *Pl'* and *B'* states reflect a corresponding reduction in pigmentation and RNA levels [13],[14]. When combined in *Pl-Rh/Pl'* or *B-I/B'* heterozygotes, alleles originally in the highly expressed *Pl-Rh* or *B-I* state heritably acquire the weak expression of *Pl'* and *B'*, respectively, and these repressed states are faithfully maintained in subsequent generations [13],[14]. This interaction between alleles on homologous chromosomes is the hallmark of a process known as paramutation [15].

Normal functions of the *nrpd1*, *rmr1* and *mop1* loci are required in trans to maintain somatic repression of the *Pl'* and *B'* states [10],[16],[17]. Although *B'* states are always meiotically transmitted [18], recessive mutations identifying individual *nrpd1*, *rmr1*, and *mop1* loci allow *Pl'* states to heritably revert to *Pl-Rh* at different frequencies [10],[11],[16],[17]. By tracking the behavior of individual *Pl1-Rhoades* alleles transmitted from plants of *Pl'/Pl-Rh* genotypes, it appears as though only NRPD1 and MOP1/RDR2 are required to mediate the allelic interactions needed to acquire a *Pl'* state [10],[11],[16].

NRPD1, RMR1, and MOP1/RDR2 function in a presumed RNA-directed DNA methylation (RdDM) pathway that produces 24 nt small RNAs and maintains cytosine methylation patterns of loci represented by those small RNAs, many of which are repetitive elements [9],[11],[19]. Individual *nrpd1*, *rmr1*, and *mop1* mutants display reductions of 24 nt siRNAs levels [9],[11],[19] and hypomethylation of cytosines at a repetitive feature 5′ of the *Pl1-Rhoades* promoter [11]. However, no methylation differences have been observed in this region between the *Pl-Rh* and *Pl'* states [11], and RMR1 is not required to mediate the allelic interaction necessary to acquire a *Pl'* state [11]. These results indicate that an RdDM-type pathway is not the causative mechanism directing paramutation in maize. The role of NRPD1 and a presumed Pol IV RNAP in effecting paramutation thus remains unclear.

Here we show that the *required to maintain repression7* (*rmr7*) locus encodes a protein related to the second largest subunit (NRPD2a) of *Arabidopsis* Pol IV and Pol V. It is unclear whether this presumed subunit ortholog functions exclusively in a maize Pol IV complex because multiple NRPD2-encoding loci were identified in the genome of maize and other grasses. The loss of 24 nt small RNAs in *rmr7* mutants parallels the phenotype of maize *nrpd1* mutants [9] indicating that this protein is necessary for functions ascribed to Pol IV. However, additional genetic and molecular comparisons between *rmr7* and *nrpd1* mutants indicate that alternative NRPD1-containing complexes with non-overlapping functions are required for the maintenance of heritable epigenetic information in maize.

## Results

### Recessive *rmr*-type mutations define the *rmr7* locus

Because plants that are homozygous for *Pl'* states exclusively have weak pigmentation patterns, darkly pigmented mutants are easy to identify. In homozygous condition, all *rmr*-type mutations phenocopy *Pl-Rh* homozygotes relative to *Pl'/Pl'* siblings [10],[17]. In an ongoing genetic screen for ethylmethane sulfonate (ems)-induced *rmr*-type mutations [10],[17], we identified three single locus recessive mutations (ems9750, ems98939, and ems062905) that failed to complement each other but which all complemented mutations defining the previously characterized *nrpd1* (*rmr6*), *rmr1*, and *mop1* loci (Protocol S1 and Table S1). Results of these genetic tests indicate that the new recessive mutations define a novel locus, provisionally designated as *rmr7*. The ems9750, ems98939 and ems062905 mutations identify the *rmr7-1*, *rmr7-2* and *rmr7-3* alleles respectively.

To begin the evaluation of *rmr7* defects on *Pl1-Rhoades* behaviors, darkly pigmented individuals homozygous for each of the *rmr7* mutant alleles were crossed to *Pl'/Pl'* plants. All 22 F_1_ plants derived from a total of two crosses with *rmr7-1*/*rmr7-1* parents had a clear Pl'-like anther phenotype (variegated pigment). Similarly, all 12 F_1_ plants derived from a cross with a *rmr7-2*/*rmr7-2* parent had variegated anthers. Three *rmr7-3*/*rmr7-3* individuals were crossed to a total of 11 different *Pl'/Pl'* plants and all 176 F_1_ plants had Pl'-type anthers. These data indicate that the *Pl1-Rhoades* alleles transmitted from these homozygous *rmr7* mutants are not recalcitrant to subsequent paramutation in the next generation and they confirm that the identified *rmr7* mutations define recessive alleles.

### The *rmr7* locus maps to the distal half of 2S

The *rmr7* locus was located to the distal half of the short arm of chromosome 2 (2S) using B-A translocations to induce segmental monosomic progeny (see Protocol S1) [20]. Plants carrying *rmr7-1* in either homozygous or heterozygous combination were pollinated by a series of *Pl'/Pl'* plants, each heterozygous for a different B-A translocation chromosome (TB-1La, TB-2Sb, TB-4Lc, TB-5La, TB-8Lc, TB-9Sd, TB-10L19). Such crosses generate a proportion of progeny that are segmentally monoploid for the respective A segment and can therefore be used to locate recessive mutations to specific chromosome positions [20]. All segmental monoploids generated with this set of B-A translocations display 50% pollen abortion and specific monoploids often have characteristic morphological phenotypes [21]. Although such monoploids were found in the progenies of all crosses, only those generated from the TB-2Sb translocation had anther phenotypes identical to those of *rmr7-1*/*rmr7-1* homozygotes (Protocol S1 and Table S2). In total, 15 of 138 F_1_ plants derived by crossing two *rmr7-1* heterozygotes by two independent *Pl'*/*Pl'*; TB-2Sb heterozygotes had darkly pigmented anthers and all 15 had the plant phenotype and 50% pollen abortion characteristic of 2S monoploids. One putative *rmr7-1*/- monoploid (02-365-54) was self-pollinated and 15/15 progeny were fully fertile and had Pl-Rh-like anthers. These data indicate that *rmr7* maps to 2S, distal to the TB-2Sb breakpoint (∼2S.5) [20].

We tested the possibility that 2S monoploids themselves affect paramutation occurring at the *Pl1-Rhoades* allele by repeating the B-A crosses using near isogenic A632 females that were either *Pl'/Pl'* or *Pl-Rh/Pl-Rh*. We found putative 2S monoploids in all progenies yet none had phenotypes distinct from those of *Pl'/Pl'* plants (Table S3). In the absence of any dosage-sensitive compensatory effects, these results indicate there are no loci on 2S that display haploinsufficiency with regard to either establishing or maintaining the *Pl'* state.

### The *rmr7* locus encodes an NRPD2 protein

**Figure 1:**
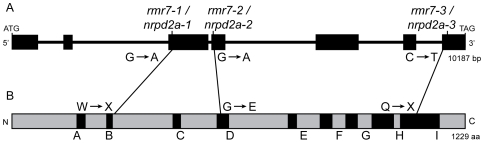
*rmr7* encodes an NRPD2-type protein and is renamed *nrpd2a*. (A) *Rmr7*/*Nrpd2a* gene model highlighting transition-type mutations in the respective mutant alleles. (B) Schematic of the predicted protein model highlighting domains (black boxes labeled A–I) that are highly conserved within all prokaryotic and eukaryotic (N)RPB2 proteins [23]. Predicted polypeptides inferred by three mutant alleles are indicated.

Given the molecular identity of MOP1 and other RMR proteins [9],[11],[12],[22], we hypothesized that *rmr7* might also encode a component in a RdDM-type pathway. A BLAST search of the maize genome sequence available on 2S identified a gene model encoding a protein with highest similarity to the second largest subunit of Pol IV and Pol V from *Arabidopsis* and containing peptide signatures representing all the conserved domains (A–I) found in similar subunits of all known RNAPs [23]. Genomic regions representing this candidate gene were amplified via polymerase chain reaction (PCR) and sequenced from homozygous mutant plants of all three ems-derived *rmr7* alleles and the non-mutant progenitor. Three single transition-type lesions within the putative coding regions of the candidate gene were identified in mutant plants with respect to both the progenitor and B73 genomic sequences (Figure 1A, Figure S1). Two of these lesions create nonsense codons (Figure 1B). The inferred peptide encoded by *rmr7-1* lacks both conserved subunit domains C–I and the metal binding sites known to be critical for *Saccharomyces cerevisiae* RNAP catalysis and *Arabidopsis* Pol IV/V function [23],[24]. The inferred peptide encoded by the *rmr7-3* allele lacks the conserved domains that are required for contacts with the largest subunit in *S. cerevisiae* Pol II [23]. The lesion identified in *rmr7-2* predicts an amino acid substitution of a glycine residue that is strictly conserved amongst all RNAP second largest subunits to glutamate (Figure 1B, Figure S1). These three lesions strongly indicate that *rmr7* encodes an NRPD2-type protein hereafter referred to as NRPD2a. Correspondingly, the *rmr7* locus and mutant alleles are renamed *nrpd2a*, *nrpd2a-1*, *nrpd2a-2* and *nrpd2a-3*, respectively.

**Figure 2:**
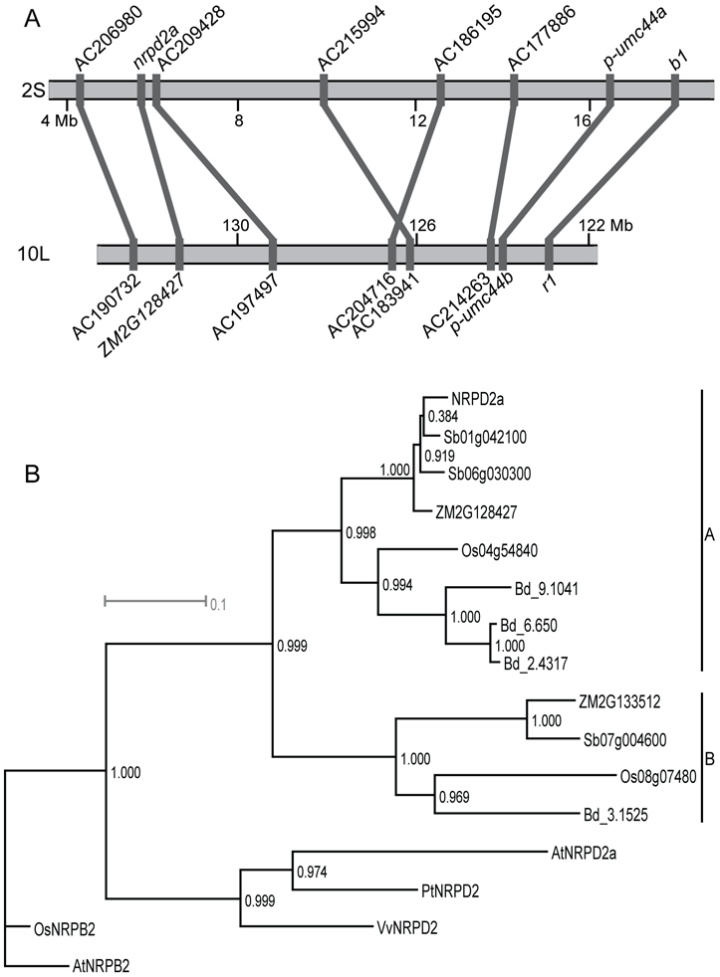
Grasses contain multiple NRPD2-type proteins. (A) *nrpd2a* and *ZM2G128427* are located on homoeologous chromosome regions of 2S and 10L. Gray boxes represent maize BACs, simple sequence repeat markers, or genes. Gray lines connect homoeologous features. Chromosomes are anchored on the right by the known homoeologous gene pair of *b1* and *r1* [25]. (B) Maximum likelihood tree produced from alignment (Figure S1) of NRPD2a with other plant NRPD2-like proteins. Grasses maize (prefix: ZM), sorghum (Sb), rice (Os), and *Brachypodium distachyon* (Bd) have multiple NRPD2-like proteins compared to eudicots poplar (Pt), grape (Vv), and *Arabidopsis* (At). *Arabidopsis* contains an additional pseudogene, *nrpd2b*, which is weakly expressed and does not produce a full-length protein (not shown) [33]. NRPB2 proteins from rice and *Arabidopsis* root the tree. Outgroup branch length is not to scale.

BLAST searches identified two additional NRPD2-encoding gene models in the maize genome, *ZM2G133512* on 10S and *ZM2G128427* on 10L. 2S and 10L contain duplicated regions retained from an ancient tetraploidy event in maize [25]. We identified synteny between the chromosomal regions around *nrpd2a* and *ZM2G128427* (Figure 2A) indicating these genes are homoeologs. No significant synteny was observed between the regions around *nrpd2a* and *ZM2G133512*. Both *ZM2G133512* and *ZM2G128427* are predicted to encode full-length proteins with high amino acid sequence conservation to that encoded by *nrpd2a* (67 and 94% identity, respectively) indicating that these loci likely produce functional NRPD2-type proteins.

To determine if the expansion of genes encoding NRPD2-type proteins was unique to maize, we identified full-length predicted proteins from other plant genomes including the eudicots grape and poplar and the grasses *Brachypodium distachyon*, rice, and sorghum. These protein sequences were aligned (Figure S1), and a maximum likelihood tree was constructed (Figure 2B) using the second largest subunit of Pol II, NRPB2, from *Arabidopsis* and rice as outgroups. This analysis indicates that retention of duplicated genes encoding NRPD2 proteins has occurred in grasses but not eudicots. Within the grasses, the NRPD2-type proteins fall into two distinct clades. Clade B contains single NRPD2-type proteins from each grass species while loci in clade A have undergone further duplications, one in maize and sorghum and two in the *Brachypodium* lineage (Figure 2B). This diversity of NRPD2-type subunits implies that, in contrast to *Arabidopsis*, Pol IV and Pol V-type RNAPs in the grasses may not be defined by a shared second largest subunit.

### NRPD2a is required to maintain genome-wide small RNA levels but not RNA transcripts from *CRM* retrotransposons

**Figure 3:**
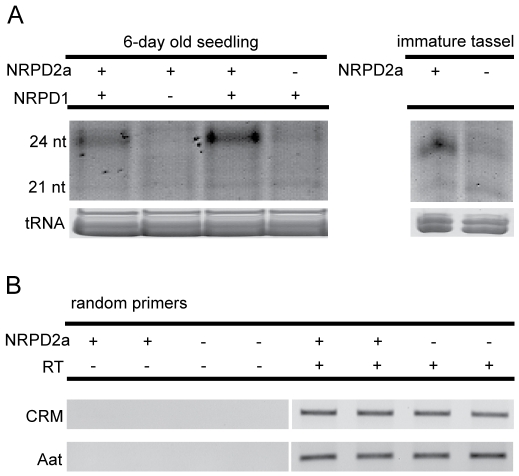
Molecular profiles of NRPD2a action. (A) EtBr-stained PAGE gel of small RNAs isolated from 6-day old seedlings and immature tassels of *nrpd2a-1* homozygotes, *nrpd1-1* homozygotes, and their respective non-mutant siblings. (B) PCR amplicons from *CRM2* LTRs and the *Aat* gene generated from total RNAs that were random primed and treated without (−) or with (+) reverse transcriptase (RT). RNAs were isolated from 4-day old seedlings of either *nrpd2a-1* homozygotes (dark seedling tissues) or heterozygous siblings (near colorless seedlings).

Mutations in *nrpd1* reduce 24 nt RNA abundances to approximately 18% of non-mutant levels [9] in immature cobs. Similar profiles assessed on 6-day old seedlings show that the 24 nt RNA class is reduced to similar levels in both *nrpd1* and *nrpd2a* mutants (14% and 15%, respectively) (Figure 3A) and parallel profiles are seen in tissues from immature tassels (Figure 3A). These results are consistent with the interpretation that both NRPD1 and NRPD2a are required for Pol IV function. No other NRPD2-type protein appears to compensate for the loss of NRPD2a function and hence this locus appears to provide the sole functional NRPD2-type protein utilized during early seedling and tassel development. This result validates the assignment of *rmr7* as encoding a functional NRPD2-type protein.

Previously, we found that individual components of the RdDM pathway have different effects on the accumulation of *CRM* Long Terminal Repeat (LTR)-type retrotransposon transcripts. In 4-day-old seedlings, the subclass of *CRM2-*type transcript levels are increased ∼6-fold in *nrpd1* mutants while they are diminished or eliminated in both *rmr1* and *mop1*/*rdr2* mutants [26]. However, identical semi-quantitative RT-PCR analyses indicate that loss of NRPD2a has no obvious effect on the accumulation of *CRM2*–derived LTR RNAs in 4-day old seedlings (Figure 3B). Densitometry measurements of ethidium bromide-stained bands from both prior results [26] and from Figure 3B indicate that the ratios of *CRM2-*derived LTR RNAs to *Aat* transcripts are 4.2 and 0.86 (+/−0.24 s.e.m.) in *nrpd1* and *nrpd2a* mutants, respectively. Quantitative RT-PCR analyses confirmed that the relative levels of *CRM2* RNA normalized to Pol II-derived *Aat* transcripts are similar in *nrpd2a-1* mutants and heterozygous siblings (Protocol S1 and Figure S2). Applying two-sample z-test statistics to compare average ratios −/+ NRPD1 [26] and -/+ NRPD2a (Figure S2) of *Aat-*normalized *CRM2* LTR RNA levels, there is a significant effect of the *nrpd1-1* mutation (z = 3.35, p<0.001) but not the *nrpd2a-1* mutation (z = −0.35, p = 0.73) on *CRM2* LTR RNA levels. These results indicate that while the NRPD2a subunit is necessary for 24 nt RNA accumulation, its absence does not completely mimic the loss of NRPD1.

### NRPD2a is not required to maintain the meiotically-heritable *Pl'* state

The *Pl'* state can revert to a meiotically transmissible *Pl-Rh* state when *Pl1-Rhoades* is in hemizygous condition [27],[28] or in all homozygous *rmr* mutants evaluated to date [10],[17]. *Pl1-Rhoades* alleles of *Pl-Rh* state are often sexually transmitted from *rmr1*, *required to maintain repression2*, or *nrpd1* mutant plants even though *Pl1-Rhoades* alleles of *Pl'* state were originally introduced into those mutants. Given that *nrpd2a* mutant plants—generated by either selfing *Pl'*/*Pl'*; *Nrpd2a*/*nrpd2a* plants, or by intercrossing *Pl'*/*Pl'*; *Nrpd2a*/*nrpd2a* and *Pl1-Rhoades*/*Pl1-Rhoades*; *nrpd2a*/*nrpd2a* siblings—display a plant phenotype indistinguishable from that of *Pl-Rh*/*Pl-Rh* plants (Anther Color Score of 7; [13]), we expected that some *Pl1-Rhoades* alleles would be transmitted in Pl-Rh-like state from such plants. This expectation was not met as crosses between homozygous *nrpd2a* mutants and a series of *Pl-Rh*/*Pl-Rh* tester stocks gave rise to progenies exclusively showing anther phenotypes typical of *Pl'/Pl'* genotypes (Anther Color Scores of 1—4; [13] Table 1). Thus, similar to the comparisons made with *CRM2*-derived RNAs, NRPD2a defects are unlike those of NRPD1 with regards to maintaining the meiotically-heritable feature specific to paramutant *Pl'* states [10].

**Table 1:** *Pl'* does not revert to *Pl-Rh* in *nrpd2a* mutants.

Allele	No. progeny sets	No. different *Pl-Rh* testers	ACS1	ACS2	ACS3	ACS4	ACS5-7
*nrpd2a-1*	12	7	119	100	12	4	0
*nrpd2a-2*	4	1	25	39	0	5	0
*nrpd2a-3*	1	1	5	17	0	2	0

Number of progeny with specific anther color scores (ACS) from the indicated number of *Nrpd2a/Nrpd2a; Pl-Rh/Pl-Rh* X *nrpd2a*/*nrpd2a*; *Pl′*/*Pl′* testcross progeny sets are given for specific *nrpd2a* alleles.

Additional tests were made with pollen from a single *nrpd2a-2* homozygote crossed to *Pl-Rh/Pl-Rh* and *Pl'/Pl'* half siblings (A632 *Pl-Rh/Pl-Rh* and spontaneously arising *Pl'/Pl'* siblings crossed to a W23 *Pl-Rh/Pl-Rh* stock). Both progenies consisted of individuals of exclusively Pl'-like phenotypes (28 and 27 individuals, respectively). Collectively, these results indicate that *Pl1-Rhoades* alleles are able to maintain some meiotically-heritable feature in *nrpd2a* mutants that allows them to retain the ability to facilitate paramutation in the next generation. However, following 5 generations of inbreeding via single seed descent, a similar *Pl-Rh/Pl-Rh* testcross using an A619 *Pl-Rh/Pl-Rh* stock gave rise to a progeny set that had 5 of 13 individuals displaying phenotypes typical of *Pl-Rh/Pl-Rh* plants indicating that reversion of *Pl'* to *Pl-Rh* can occur following multiple generations of conditioning in the absence of NRPD2a function.

### NRPD2a is required to acquire a heritable paramutant *B'* state

**Figure 4:**
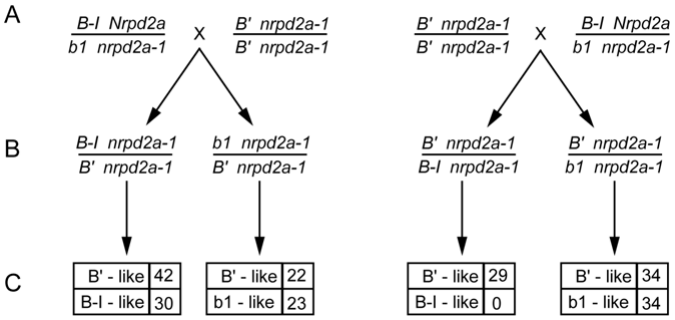
NRPD2a is required for acquisition of a *B'* state. (A) One *B-I Nrpd2a*/*b1 nrpd2a-1* plant was reciprocally crossed to one *B' nrpd2a-1*/*B' nrpd2a-1* plant to combine *B-I* and *B'* in *nrpd2a-1* homozygous mutants. (B) Resulting dark, B-I-like F_1_ progeny genotypes, including both non-recombinant and recombinant types. (C) Numbers of progeny plants with the indicated color phenotypes resulting from testcrosses between thirteen F_1_ plants (B) and colorless *b1 Nrpd2a*/*b1 Nrpd2a* plants. Results from individual testcrosses can be found in Table S4.

In *B-I*/*B'* heterozygotes, the strongly expressed *B-I* state invariably changes to a transcriptionally repressed *B'* state [14]. To ask whether *B-I* would acquire a *B'* state in the absence of NRPD2a function, *B-I* and *B'* states were combined in *nrpd2a-1* homozygotes (Figure 4A and 4B) and then the *B1-I* alleles were evaluated for plant pigmentation function following transmission to recessive *b1* allele testers (Figure 4C). Reciprocal crosses between a single *B-I Nrpd2a*/*b1 nrpd2a-1* plant and a single *B' nrpd2a-1* homozygote were used to generate the progeny plants in which *B-I* and *B'* states were combined. As expected, pigment phenotypes of *nrpd2a-1*/*nrpd2a-1* individuals were identical in both *B'/B-I* and *B'*/*b1* genotypes (Figure 4B). Based on the visual phenotypes of the test cross progenies, only *B'* states are transmitted from *B' nrpd2a-1*/*b1 nrpd2a-1* plants (Figure 4C; Table S4). This result indicates that NRPD2a is not required to maintain the meiotically-heritable *B'* state and is in accord with similar tests of the effects of NRPD2a on the maintenance of *Pl'* states (Table 1). When *B1-I* alleles were transmitted from four individual *B-I nrpd2a-1*/*B' nrpd2a-1* plants, both B'-like and significantly darker B-I-like test cross progeny phenotypes were found in approximately equal numbers (Figure 4C, Table S4). Since *B-I* invariably changes to *B'* in *B-I/B'* heterozygotes [14], this exceptional result indicates that paramutation of *B-I* to *B'* in a *B-I*/*B'* heterozygote depends on NRPD2a function. However, *B1-I* alleles transmitted from two individual *B' nrpd2a-1*/*B-I nrpd2a-1* plants gave exclusively B'-like testcross progeny phenotypes (Figure 4C; Table S4) indicating just the opposite; that paramutation is not dependent on NRPD2a function. Interestingly, the difference between these two contrasting results correlated with the parent of origin for the *B'* state. When *B'* was transmitted through the homozygous *B' nrpd2a-1* female, paramutation occurring within the subsequent *B' nrpd2a-1*/*B-I nrpd2a-1* progeny was not prohibited, yet when *B'* was contributed from the homozygous *B' nrpd2a-1* male, paramutation appeared to require NRPD2a function. These results indicate NRPD2a is conditionally required to acquire the *B'* state and stand in contrast to those of prior tests in which NRPD1 was found to be required for *B-I* to change to a *B'* state in *B'*/*B-I*, homozygous *nrpd1* mutants, even though *B'* had been maternally transmitted to those mutants [10].

### The *nrpd2a* mutations do not affect plant development

In sharp contrast to NRPD1 defects [9],[29], the Pl-Rh-like phenotype seen in homozygous *nrpd2a-1* plants is not associated with any obvious developmental abnormalities. Field observations of F_2_ families segregating the *nrpd2a-1* mutation as well as comparison of *nrpd2a-1*/*nrpd2a-1* and *nrpd2a-1*/*Nrpd2a* individuals derived from intercrossing *nrpd2a-1/nrpd2a-1* and *nrpd2a-1*/*Nrpd2a* F_5_ siblings show relative uniformity of plant morphology. Moreover, all four S_4_ lines of *nrpd2a-1* homozygotes derived by single seed decent had excellent survivorship (77/80 seeds gave fertile plants) and had morphologically normal plants. This stands in contrast to the strong degradation of plant quality documented for NRPD1 mutants [29] that rarely produce any seed past the S_3_ generation or any morphologically normal plants past the S_2_ generation.

Similarly, field observations of F_2_ families segregating the *nrpd2a-2* mutation as well as comparison of *nrpd2a-2*/*nrpd2a-2* and *nrpd2a-2*/*Nrpd2a* individuals derived from intercrossing *nrpd2a-2/nrpd2a-2* and *nrpd2a-2*/*Nrpd2a* F_2_ siblings show similar uniformity of type. Height measurements over 3 independent progeny sets show that *Nrpd2a*/*nrpd2a-2* plants average 71.4 cm +/− 1.1 (s.e.m.; n = 24) whereas homozygotes average 68.3 cm +/− 1.1 (s.e.m.; n = 25). These measurements are not statistically different from one another (2-sample z-test; z = 2.0; P>0.05) and there are currently no other compelling observations to indicate that the locus defined by either the *nrpd2a-1* or *nrpd2a-2* mutations is required for normal growth and development. These results parallel those of the *CRM2* RNA abundances and those measuring the effects of NRPD2a on paramutations at the *pl1* and *b1* loci in indicating that the functions of maize NRPD2a and NRPD1 do not completely overlap.

## Discussion

Genetic screens in maize have identified two RNA polymerase subunits as required to maintain repressed epigenetic states associated with paramutation in maize. Previously, we identified *rmr6/nrpd1* as encoding NRPD1, the largest subunit of Pol IV [9], and here we report the identification of *rmr7/nrpd2a* as encoding NRPD2a, the second largest subunit of Pol IV and, potentially, Pol V. Derived from Pol II, Pol IV and Pol V are functionally distinct RNAPs defined by their largest subunits, NRPD1 and NRPE1, respectively [1]. The catalytic cores of these respective polymerases are created by physical interaction between the largest and second largest subunits (NRPD2/NRPE2). In *Arabidopsis*, Pol IV and V share a single second largest subunit, AtNRPD2a. Additional subunits are shared with Pol II, or exist in Pol IV and/or Pol V-specific forms [30]. *nrpd2a* is one of three maize loci predicted to encode a protein similar to AtNRPD2a. Based on predicted protein alignments with *S. cerevisae* RPB2, the additional maize NRPD2-type proteins are predicted to be functional. Further, all three *nrpd2*-type loci appear to express RNA more or less constitutively throughout growth and development [31]. This diversity of potentially functional NRPD2-type proteins is conserved throughout other grass species but not the representative eudicots.

Previous phylogenetic analysis concluded that NRPD2 derived from a single duplication of RPB2 in the ancestor of land plants [1]. Our analysis of angiosperm NRPD2 sequences from complete or near complete genomes indicates that multiple *nrpd2* locus duplications have occurred after the divergence of monocots and eudicots. *Arabidopsis* is the only representative eudicot with evidence of a *nrpd2* locus duplication, yet only one functional locus has been retained from this recent event [3],[4],[32],[33]. An *nrpd2* locus duplication in the grass common ancestor resulted in two distinct and well-supported clades, A and B. Unlike *Arabidopsis*, all of these *nrpd2-*encoding loci appear to be functional. The relative timing of this duplication corresponds with a whole genome duplication that occurred in the cereal genome prior to the divergence of rice, *Brachypodium*, sorghum, and maize [34]. Accordingly, the rice clade A (Os04g54840) and clade B (Os08g07480) loci are located in the homoeologous r8-r4 chromosomal segments retained from this duplication [35].

Within clade A, further *nrpd2* duplications have been retained in individual species lineages. The two maize clade A loci, homoeologs *nrpd2a* and *ZM2G128427*, are located in regions syntenic with sorghum chromosome 6 [35], the location of clade A locus Sb06g030300. However, the additional sorghum clade A locus, Sb01g042100, is in an asyntenic region on chromosome 1 [35] indicating that the duplication in sorghum occurred independently of that in maize. The maize clade A duplication is consistent with a tetraploidy event which occurred after the divergence of maize and sorghum [36],[37] while the sorghum clade A duplication corresponds to a small-scale event occurring post-divergence [37]. The origins of the *Brachypodium* duplications are unclear, as no large scale duplications have been proposed in that lineage, but the high degree of amino acid similarity (98.5%) between Bd_6.650 and Bd_2.4317 indicates that this duplication was relatively recent.

While both the eudicot and grass lineages have undergone genome duplication events [38], only the grasses have retained potentially functional NRPD2-type duplicates. This general observation indicates that grasses have a fundamentally different type of polymerase biology relative to eudicots. One possibility is that the additional NRPD2-type proteins interact with both Pol IV and Pol V, as in *Arabidopsis*, but in a semi-redundant fashion. Complete functional redundancy is inconsistent with recessive loss-of-function lesions at the *nrpd2a* locus, but perhaps the individual NRPD2-type subunits overlap only for certain RNAP functions. Alternatively, the A and B clades identified in the phylogenetic tree could represent a functional division between NRPD2 proteins that participate in either Pol IV, Pol V or in RNAPs that are specific for different tissues or developmental time points. Regardless, the grasses clearly support a potentially greater diversity of RNAP complexes than the representative eudicots examined here.

Functional analyses of *nrpd2a* mutations are consistent with the idea that grasses have a greater diversity of functional RNAPs than those found in *Arabidopsis*. Like NRPD1, NRPD2a is required for somatic maintenance of *Pl'* states and approximately 85% of all 24 nt RNA accumulation, consistent with a Pol IV-type function. However, loss of NRPD2a function does not completely mimic the loss of NRPD1 as *nrpd2a* mutants have unique molecular, genetic, and morphological phenotypes. These contrasting results indicate that NRPD2a is required for only a subset of presumed Pol IV functions and supports the hypothesis that maize, and perhaps other grasses, utilize functionally distinct Pol IV-type RNAPs defined by a shared NRPD1 together with one or the other NRPD2-type subunits.

Although the DNA-dependent RNA polymerase responsible for the levels of *CRM2* transcript seen here remains unknown, we have previously shown that these non-polyadenylated *CRM2* RNA levels are decreased in both *rmr1* and *rdr2* mutants, thereby indicating that they are primarily products of an RNA-dependent RNA polymerase [26]. We have proposed that the increased poly-adenylated *CRM2* RNAs observed in *nrpd1* mutants are due to increased Pol II transcription in the absence of Pol IV competition for the *CRM2* LTRs [26]. In non-mutant conditions, Pol IV facilitates repression of *Pl1-Rhoades* by inhibition of Pol II, and we have hypothesized that this interference occurs either through direct competition for initiation sites or by titration of shared RNAP subunits [9]. The results presented here indicate that there are functionally distinct Pol IV-type RNAPs, those that require NRPD2a (for 24 nt RNA accumulation) and those that do not (for inhibition of Pol II). Either one or the other NRPD2-type proteins define these functionally distinct complexes or perhaps NRPD1 can act independently of a RNAP holoenzyme.

A gain-of-function *nrpd2a* mutation that could dominantly interfere with all NRPD1-containing complexes would be predicted to have phenotypic overlap with *nrpd1* mutants. While no such dominant alleles have been identified in our mutational screens (0/15,000 M_1_ plants), Sidorenko *et al.* [31] report on a semi-dominant mutant allele (*Mop2-1*) identifying the same locus as *nrpd2a* that predicts a single amino acid change in the terminal domain presumably required for interaction with NRPD1. Since our evaluation of 2S segmental aneuploids indicate that the *nrpd2a* locus is haplosufficient, the dominant nature of the *Mop2-1* allele is unlikely to be simply due to a dosage effect. Homozygous *Mop2-1* mutants do have a developmental phenotype reported to be similar in some respects to that displayed by *nrpd1* mutants [31] and this may indicate that the NRPD2a variant encoded by *Mop2-1* poisons multiple RNAP complexes.

While the identification of NRPD2a has highlighted the additional RNAP complexity of maize relative to *Arabidopsis*, it is still unclear how a presumed Pol IV ortholog, and the greater RdDM machinery, affects paramutation. Several distinct but overlapping conceptual functions are required for paramutation including 1) acquisition of a repressed epigenetic state through unknown trans-homolog interactions, 2) somatic maintenance of such acquired repressed states, and 3) meiotic transmission of paramutant states [10]. As our forward genetic screens specifically looked for failure to maintain repressed *Pl'* states somatically, all RNAP and RdDM factors identified to date are required for this function. However, with pedigree analyses of mutant materials, it is clear that these individual factors have different effects on the acquisition and heritable maintenance of paramutant states [10],[11],[16],[17].

Functionally distinct Pol IV-type complexes might help explain why NRPD2a has a different phenotype from NRPD1 with regard to meiotic transmission of *Pl'* and the acquisition of *B'* states. Since NRPD2a is required for 24 nt RNA accumulation, the small interfering class of small RNAs (siRNAs) do not appear to be essential for the meiotic transmission or acquisition of paramutant states, although cumulative loss of siRNAs over several generations might result in failure to maintain meiotic transmission. Similarly, RMR1, which is also required for siRNA production, is not required for the allelic interaction required to acquire paramutant states even though it is required to some extent for the meiotic transmission of paramutant states [11]. NRPD1 is required for both siRNA production and meiotic transmission of paramutant *Pl'* states but its role in repressing Pol II activity may be the more critical function with regard to the acquisition of paramutant states. The apparent conditional requirement of NRPD2a for the establishment of *B'* states could indicate that an NRPD2a-containing RNAP is necessary in pollen to successfully convert *B-I* to *B'*. Alternatively, the parent of origin effect we observed could be due to somatic mosaicism with spontaneous change of the *B-I* allele to *B'* in the soma giving rise to the apical tassel but not to the lateral ear shoot, or perhaps the *nrpd2a-1* mutation imparts a semi-dominant effect that was manifest in the relatively small sample size evaluated here. While such behaviors remain a possibility, we have not observed these types of effects in similar tests of *B'* establishment [10].

From the analyses of maize and *Arabidopsis* mutants, it is clear that the evolution of Pol IV and Pol V-type RNAPs facilitated unique mechanisms for epigenetic repression in plants. While models for Pol IV and Pol V function have been generated in *Arabidopsis*, it will be important to determine how applicable they will be in the cereal crops. The inferred increased diversity of RNAPs combined with enormous expansion of repetitious sequences in large genome cereals provides a potential basis for the innovation of regulatory novelty. A further understanding of the mechanistic relationship between paramutation and maize RNAP diversity promises to illuminate how such features have been co-opted during evolution and domestication of the grasses.

## Materials and Methods

### Nomenclature

The maize Pol IV largest subunit has been designated “RPD1” in two prior publications from this laboratory. The addition of the letter “n” to all plant RNA polymerase loci, genes, and proteins was substituted during the production process.

Following standard conventions (http://www.maizegdb.org/maize_nomenclature.php), maize loci are designated in lower-case italics (i.e. *pl1*). Specific recessive alleles are designated with a dash, followed with a descriptor of the allele, usually the inbred line from which the allele originated (i.e. *pl1-B73*). Dominant alleles begin in uppercase lettering (i.e. *Pl1-Rhoades*). Translocation breakpoints are indicated with a “T” and paramutagenic states with a prime symbol (*'*). Plant phenotypes displayed by particular states of *Pl1-Rhoades* are written in non-italic text (i.e. Pl'). All diploid genotypes are presented with pistillate (female)-derived factors first and staminate (male)-derived factors second.

### Genetic analyses

Hand pollinations were used for all crosses. *Pl1-Rhoades* expression was visually assessed for each progeny individual as described [13].

### Candidate gene sequencing

BLAST searches identified sequence similar to *Arabidopsis nrpd2a* on maize BAC c0009N09 (Genbank accession AC191113) on chromosome 2S, and a gene model was predicted using maize EST sequence (Genbank accession AY104560) and FGENESH+. Oligonucleotide primers spanning the predicted coding region (Table S5, Sigma-Genosys, www.sigmaaldrich.com/Brands/Sigma_Genosys.html) were designed from this sequence and used in PCR reactions with genomic DNA isolated from plants homozygous for the three *nrpd2a* mutant alleles and the non-mutant progenitor line. PCR amplicons were purified using the QIAquick gel extraction kit (Qiagen, www.quiagen.com) and at least two independent amplicons were dideoxy sequenced (UC Berkeley DNA Sequencing Facility, mcb.berkeley.edu/barker/dnaseq/). Intron/exon boundaries were verified by amplification from cDNA and subsequent sequencing. cDNA sequences can be retrieved from Genbank using accession numbers GQ356034 through GQ356037.

### Phylogenetic analysis

NRPD2-type sequences from diverse plant lineages were obtained through tblastn searches of Phytozome (www.phytozome.net) and BrachyBase (http://blast.brachybase.org/) using ZmNRPD2a or AtNRPD2a as queries. When necessary, gene models were predicted with FGENESH+ (www.softberry.com) using similarity to either ZmNRPD2a or AtNRPD2a. Some sequence names were altered for clarity in Figure 2. Full-length names are as follows: AtNRPB2 = At4g21710, OsNRPB2 = Os03g44484, AtNRPD2a = At3g23780.1, PtNRPD2 = XP_002324332, VvNRPD2 = XP_002283296, for maize sequences add GRM to the beginning of the sequence name, for *Brachypodium* sequences substitute super for Bd. Sequences were aligned using MAFFT (http://align.bmr.kyushu-u.ac.jp/mafft/online/server/) and a maximum likelihood tree was constructed with Phyml (http://mobyle.pasteur.fr/cgi-bin/portal.py) using the JTT amino acid substitution model and NNI+SPR tree topology search operation. The tree was edited with Dendroscope 2.2.2 [39]. The alignment was edited using GeneDoc 2.6.04 as previously described [9]. Homoeologous regions were identified using ESTs, simple sequence repeat markers, and genes to identify sequence similarity on chromosomes 2S and 10L. Features used and the corresponding BACs they identify are as follows: AY111545 (AC206980, AC190732), *nrpd2a* (AC191113), *ZM2G128427* (AC199156), AY112227 (AC209428, AC197497), AY110965 (AC215994, AC204716), AY105682 (AC186195, AC183941), AY109473 (AC177886, AC214263), *p-umc44b*, *p-umc44a*, *b1* (AC191025), *r1* (AC199387).

### Small RNA abundance analysis

Sibling mutant and non-mutant whole seedlings were identified by tissue coloration 6 days post-imbibition and pooled for RNA extractions. Tassel branches were collected prior to anthesis from mutant and non-mutant siblings and used for RNA extractions. Small RNA fractions were enriched from total RNA and visualized following polyacrylamide gel electrophoresis (PAGE) as previously described [9]. Relative 24 nt RNA abundances were quantified by normalizing to 21 nt RNAs as previously described [9].

### RT–PCR

Total RNA was extracted from whole seedlings 4 days post-imbibition by Trizol (Invitrogen) purification. Oligo(dT)-primed cDNA was generated as previously described [9]. Random primed cDNA was generated using 1 µg of total RNA that was reverse transcribed with the Superscript III enzyme (Invitrogen) in the presence of 250 pmol of random hexamers in a 20 µL reaction. *CRM2* LTR cDNA sequence was then amplified via PCR with previously described primers [40]. The *alanine aminotransferase* (*Aat*) cDNA was PCR amplified using previously described primers [9]. The PCR program used is as follows, repeated 30 times: 94°C for 30 sec, 57°C for 30 sec, and 72°C for 45 sec. Relative *CRM2* abundances were quantified by normalizing to *Aat* RNAs as previously described [9].

## Supporting Information

Figure S1 Alignment of full-length NRPD2 proteins from diverse plant species. Full-length NRPD2 proteins were aligned using MAFFT and edited using GeneDoc. Shading represents 60% (lightest), 80%, and 100% (darkest) sequence conservation. Conserved domains are highlighted below the text, and NRPD2a lesions are indicated by underlined text.
(0.39 MB PDF)

Figure S2 qRT-PCR analysis. qRT-PCR analysis of the relative abundance of *CRM2* LTR RNAs in homozygous *nrpd2a-1* mutants relative to heterozygous siblings (error bars are ±1 s.e.m.).
(0.17 MB DOC)

Table S1 ems9750, ems98939, and ems062905 define the *rmr7* locus. Progeny anther color scores from crosses between ems9750 (*rmr7-1*), ems98939 (*rmr7-2*), and ems062905 (*rmr7-3*) mutants and plants carrying other *rmr* and *mop* mutations. *rmr6* is also known as *nrpd1*.
(0.06 MB DOC)

Table S2 *rmr7* maps distal to the TB-2Sb breakpoint on 2S. Progeny of independent crosses between *Rmr7*/*rmr7-1* and *Pl'*/*Pl'*; TB-2Sb heterozygotes resulted in 15 individuals with dark anthers (ACS scores 5–7). All dark plants showed characteristic 50% pollen abortion and plant phenotypes of 2S monoploids.
(0.03 MB DOC)

Table S3 2S monoploids do not affect paramutation. Progeny anther color scores for crosses between A632 *Pl1-Rh*/*Pl1-Rh* and *Pl'*/*Pl'*; TB-2Sb heterozygotes.
(0.04 MB DOC)

Table S4 Evaluation of *b1* paramutation occurring in individual *nrpd2a-1* F1 homozygotes through crosses to homozygous *Nrpd2a b1* testers. Two plants, one *b1 nrpd2a-1*/*B-I Nrpd2a*; *Pl'*/*Pl'* and one *B' nrpd2a-1*/*B' nrpd2a-1*; *Pl'*/*Pl'*, were reciprocally crossed to generate the respective 02–939 (*B' nrpd2a-1* homozygote male) and 02–940 (*B' nrpd2a-1* homozygote female) progeny sets. Individual F1 progeny plants having dark plant and anther colors were crossed to *b1 Nrpd2a*/*b1 Nrpd2a*; *Pl-Rh*/*Pl-Rh* testers and the number of testcross progeny plants having a colorless (*b1*/*b1*), B'-like (*b1*/*B'*), or B-I-like (*b1*/*B-I*) phenotype are tallied.
(0.05 MB DOC)

Table S5 Oligonucleotide primers used for *nrpd2a* sequencing. Oligonucleotide primers designed to amplify the predicted coding regions of the *nrpd2a*-like gene candidate from genomic DNA sequence by PCR.
(0.04 MB DOC)

Protocol S1 Additional materials and methods used for genetic analyses.
(0.03 MB DOC)
