# Supplementary material for: Diversity of Pol IV Function Is Defined by Mutations at the Maize rmr7 Locus
Source: PLoS Genet. 2009 Nov 20;5(11):e1000706. doi: 10.1371/journal.pgen.1000706 (PMC2775721; doi:10.1371/journal.pgen.1000706)
Supplement: Figure S1 — Alignment of full-length NRPD2 proteins from diverse plant species. Full-length NRPD2 proteins were aligned using MAFFT and edited using GeneDoc. Shading represents 60% (lightest), 80%, and 100% (darkest) sequence conservation. Conserved domains are highlighted below the text, and NRPD2a lesions are indicated by underlined text. (0.39 MB PDF) [file pgen.1000706.s001.pdf]

Figure S1. Alignment of full-length NRPD2 proteins from diverse plant species.

Full length NRPD2 proteins were aligned using MAFFT and edited using GeneDoc. Shading represents 60% (lightest), 80%, and 100% (darkest) sequence conservation. Conserved domains are highlighted below the text, and NRPD2a lesions are indicated by underlined text.

```
                                    20                  40                  60
NRPD2a       -------------------------MEEPQKDSGQPSKSSDSELEA--------------    21
Sb01g04210   -------------------------MEELQKDGGQQSNGSDSEPEA--------------    21
Sb06g03030   -------------------------MEELQKDSGQPSNGSDSEPEA--------------    21
ZM2G128427   -------------------------MEELQKDSALPSNSSDSEPEA--------------    21
OsNRPD2a     -------------------------MEEPSKDNGQSSCVVDPELEP--------------    21
Bd_9.1041    -------------------------MEDIPRGSGHSTNGTEPELEP--------------    21
Bd_6.650     -------------------------MEEPPRDNAHSKNGLEPELEP--------------    21
Bd_2.4317    -------------------------MEEPPRDNAPSKKGPEPELEP--------------    21
ZM2G133512   -------------------------MGKPQTDGDQSPVNSD--TDVMDC-----------    22
Sb07g00460   -------------------------M-----DGDQSTVVLD--TDMMDC-----------    17
OsNRPD2b     MWDEKERPRVFCLAPQDGEAVWWLAMEDPEITGQQSPNATSIVTDLMDLDDIIVEGNEVQ    60
Bd_3.1525    -------------------------MEEPQANDLQSPNGSDIELDVVDF-----------    24
AtNRPD2a     -------------------------M----------------------------------     1
PtNRPD2      -------------------------M----------------------------------     1
VvNRPD2      -------------------------MGASDAKGD--------------------------     9
OsNRPB2      -------------------------MEDDEYEEG--------------------------     9
AtNRPB2      -------------------------ME---------------------------------     2


                                    80                 100                 120
NRPD2a       MVLDDNGAGKSHSMEEN----------RDSPIDVDG---------------------GQS    50
Sb01g04210   MVLDDNGAGKSHSMD----------GNRDSPVDVDE---------------------GQS    50
Sb06g03030   MVLDDNGAGKSRSMEGNEDSPIADVGKDSPIDVNE----------------------GQS    60
ZM2G128427   MELDDNGAGKSHSIEGN----------RDSPIDTDE---------------------GQP    50
OsNRPD2a     MMLDDAREGVSHTLD---------DANGHSSMDVDR---------------------GCH    51
Bd_9.1041    MILDDDGESRSRTID---------DSNEQSSMGIDS---------------------DRS    51
Bd_6.650     MILDDNEESGSHTMD---------DSN------------------------------GQS    42
Bd_2.4317    MILDDNEESRSHTMD---------DSN------------------------------GQS    42
ZM2G133512   LNFDGHGDVEDPHKE-------VAKGEQQPSGEVDE------------------------    51
Sb07g00460   LNFDGYGDVEDPRKE-------GAKGERQPSGKVDELQSTMDVDLTCIAYVEDEGNEKQN    70
OsNRPD2b     FAMDVDLRAIPSLKD---------GGHTDPLVQIPG---------------------DMS    90
Bd_3.1525    IALDYDGDAKCHIKQ-------DAKGEQQPLADADG---------------------GLS    56
AtNRPD2a     -----------------------------------------------------------P     2
PtNRPD2      ------------------------------------------------------------     -
VvNRPD2      ------------------------LGMSSSMGEKLS---------------------NGV    24
OsNRPB2      MEMEMGGHHHPHHGG------------------------------------------GYG    27
AtNRPB2      ------------------------------------------------------------     -


                                   140                 160                 180
NRPD2a       SMDVDIKGKSSLSDDVNGKSSSEPYSNAPIDMSVESLEKFCKEAS----RSFFDEVGLIS   106
Sb01g04210   SMDVDTKGKPSLNDDVNGK-SSEPYSNAPIDLSVESLEKFCKEAS----RSFFDEVGLIS   105
Sb06g03030   SMDVDIKAKSSLNDDVNGKSSSEPYSNAPIDMSVESLEKFCKEAS----RSFFDEVGLIS   116
ZM2G128427   SMDVDIKGKSSLNDDVNGKSSSEPFSNPPINMSVESLEKFCKEAS----RSFFDEVGLIS   106
OsNRPD2a     SMDT---TRSSLGDDGKGK--RDSYAQIPVDMSIPSLEKFCKEAS----RSFFDEIGLIS   102
Bd_9.1041    SMDVDMKGKSSLDGDGKGKYSSESHEEFPIDMSLTSLEKFCKEAS----RSFFDEIGLIS   107
Bd_6.650     SMDIDIEG-SSMG------------EQIPADMNLTSLEKFCKEAS----RSFFEEIGLIS    85
Bd_2.4317    SMDIDIEG-SSMG------------EQIPADMNLTSLEKFCKEAS----RSFFEEIGLIS    85
ZM2G133512   ----------------------------------QHLEKFCKEAA----RSFFSETGLVS    73
Sb07g00460   AMDVDLKEILPEEDEGKGKASSDIPSHVPVDFDVASLEKFCREAS----RSFFSATGLVS   126
OsNRPD2b     HMDVDLRVIPSLKDGG----HADPPVQVPVDKRIASLEKLCKEAS----RSFFRETRLVS   142
Bd_3.1525    PMNVDLKGIPSLEDEREVMSSSDPCVQAPIDFNVATLEKFCKEAA----RSFFSETGLVS   112
AtNRPD2a     DMDIDVKDLEEFEATTGEINLS--------ELGEGFLQSFCKKAA----TSFFDKYGLIS    50
PtNRPD2      DMDMD-------EDLMDTTNLN--------ELGKETLQSFCKKAA----SLFFDEYGLIS    42
VvNRPD2      QMEID-------DDLMGSIEID--------DLNKEYLKTFCKKVA----VSFFNEYGLIQ    65
OsNRPB2      AEEYGAVGGEEMEDE-------------EADGDAPDEEEITQEDAWAVISAYFEEKGLVR    74
AtNRPB2      --------YNEYEPE-------------PQYVEDDDDEEITQEDAWAVISAYFEEKGLVR    41
```

```
                                     200                 220                 240
NRPD2a        HQINSYNEFVSHGLQELFDSLGEVIVEPG--YDPSKKG-SGGWKHAIIKFGRVKLEKPVF    82
Sb01g04210    HQINSYNDFVSHGLQELFDSLGEVIVEPG--YDPSKKG-SGGWKHAIIKFGRVKLEKPVF   162
Sb06g03030    HQINSYNEFVSHGLQELFDSLGEVIVEPG--YDPSKKG-SGGWKHAIIKFGRVKLEKPVF   173
ZM2G128427    HQINSYNEFVSHGLQELFDSLGEVIVEPG--YDPSKKG-SGSWKHAIIKFGRVKLEKPVF    82
OsNRPD2a      HQINSYNEFVSHGLQELFDSLGEVTVEPS--YDPSNRG-PGGWRHAIIKFGRVQLEEPVF   159
Bd_9.1041     HQINSYNDFISHGLQELFDSLGEVTVEPS--YDPSKKG-PGGWRHAIIKFGKVKLQEPVF   164
Bd_6.650      HQINSYNEFISHGLQELFDSLGEVTVEPG--YDPSKKG-PGGWRHAIIKFGKVKLEEPVF   142
Bd_2.4317     HQINSYNEFVSHGLQELFDSLGEVTVEPG--YDPSKKG-PGGWRHAIIKFGKVKLEEPVF   142
ZM2G133512    HQINSYDHFVSHGLQELFDSLGEITVEPD--YDPSNK--HGAWKHATVKFGRVKLDEPVF   129
Sb07g00460    HQINSYNQFVSHGLQDLFDSLGEITVEPD--YDPSNK--SGAWKHATIKFGRVELSEPVF   182
OsNRPD2b      HQINSYNDFVSHGLQKMFDSLDEVTVEPD--YDPSKK--VGPWRHATIKFGRVELEEPVF   198
Bd_3.1525     HQINSYNDFISHGLQELIDSVGEITVEPD--YDPSKKAEAGAWRHATIKFGRVKFEEPVF   170
AtNRPD2a      HQLNSYNYFIEHGLQNVFQSFGEMLVEPS--FDVVKKK-DNDWRYATVKFGEVTVEKPTF   107
PtNRPD2       HQINSYNSFINSGLQRVFDSFGEVAVEPG--YDSSKQK-DGEWRRASVRFGKVTLDRPSF    99
VvNRPD2       HQINSFNDFIKNGIQRVFDSFGEIPVEPG--YDPSKRG-EGDWRYASVRFGKVTLERPRV   122
OsNRPB2       QQLDSFDEFIQNTMQEIVDESADIEIRPESQHNPGRQA-EFAETLHKISFGQIYLSKPMM   133
AtNRPB2       QQLDSFDEFIQNTMQEIVDESADIEIRPESQHNPGHQS-DFAETIYKISFGQIYLSKPMM   100
```

```
                                     260                 280                 300
NRPD2a        WTGK----DEGSVDFKPWHARLQNMTYASRLIVEVTIQVYS--LEKSDKSKTGND-GFVQ   216
Sb01g04210    WTGK----DEGSVDFKPWHARLQNMTYASRLIVEVNIQVYS--LEKSDKSKTGND-GFVQ   215
Sb06g03030    WTGK----DEGSVDFKPWHARLQNMTYASRLKVEVTIQVYS--LEKSDKSKTGNE-GFVQ   226
ZM2G128427    WTGK----DEVSVDFKPWHARLQNMTYASRLRVEVTIQ-------KSDKSKTGND-GFVQ   130
OsNRPD2a      WSHGC-DIDEQSLKLKPRHARLQNMTYSSKMKVEVHFQVYS--MEKSDKAKTGND-KFGY   215
Bd_9.1041     WSDKCEDKYEEALKLKPRHARLQNMTYSSKMEVEMNIQVYS--MEKSDKAKTEND-HFGH   221
Bd_6.650      WSGKI-DIDEESLKLKPRHARLQNMTYSSKMEVEVNIQVYS--MEKSDKAKTGND-HFGH   198
Bd_2.4317     WSGKI-GIDEESLKLKPRHARLQNMTYSSKMEVEVNIQVYS--MEKSDKAKTGND-HFGH   198
ZM2G133512    MLENS-DLEEQDLKFKPRHARLQKMTYASRMNVEMTVQVYI--FDTSDKAKTGKD-THVH   185
Sb07g00460    MVDNL-DLEQQDLKFKPRHARLQRMTYASRMNVEMTAQVYI--LDKSDKAKTGKD-THVH   238
OsNRPD2b      WVDNC-DLDVETLKLKPKHARLQKMTYSSKMKVEMTVQVYS--LHKSDKAKTGED-PYIQ   254
Bd_3.1525     WVEDT-ELDEHTLKLKPKHARLQNMTYSSKMFVEMTVQVYS--LMQSDKSKIGKN-PYIQ   226
AtNRPD2a      FS------DDKELEFLPWHARLQNMTYSARIKVNVQVEVFKNTVVKSDKFKTGQD-NYVE   160
PtNRPD2       WGGTS-S-DAEH-NMFPRHARLQNMTYSARMKIHVNVQVYTQTVGRSDKFKTGID-KVVQ   155
VvNRPD2       WAGES-D-GKESLNFLPRHARLQNMTYSSRMKAQVHFQVYTQKLVRSDKYKTGKDNKYVE   180
OsNRPB2       T-----EADGETATLFPKSARLRNLTYSAPLYVDVSYRVM----------KKGHD---CE   175
AtNRPB2       T-----ESDGETATLFPKAARLRNLTYSAPLYVDVTKRVI----------KKGHD---GE   142
                                Domain A
```

```
                                     320                 340                 360
NRPD2a        KRNFMNETHWIFIGLLPVMVKSNLCLLH-----SLNE-SECLFDAGGYFLVKGMEKVFIA   270
Sb01g04210    KRDFMNETHWIFIGLLPVMVKSNLCLLH-----SLKE-SECLFDAGGYFLVKGMEKVFIA   269
Sb06g03030    KRNFMNETHWIFIGLLPVMVKSNLCLLH-----SLKE-SECLFDAGGYFLVKGMEKVFIA   280
ZM2G128427    KRNFMNETHWIFIGLLPVMVKSNLCLLH-----SLKE-SECLFDAGGYFLVKGMEKVFIA   184
OsNRPD2a      KRNIINETYYINIGRLPVMVMSNLCWLH-----KLKE-SDCQFDSGGYFLIKGMEKVFIA   269
Bd_9.1041     KRDIINETHWVSVGRLPVMVNSNLCWLH-----KLGE-SDCLFDSGGYFLIKGMEKIFIA   275
Bd_6.650      KRDIINETHWVTIGRLPVMVNSDLCWLH-----KLGE-SDCLFDSGGYFLIKGMEKIFIA   252
Bd_2.4317     KRDIINETHWVTIGRLPVMVNSDLCWLH-----KLGE-SDCLFDSGGYFLIKGMEKIFIA   252
ZM2G133512    KREIMTETKQINIGLLPVMVKSNLCWLH-----KSQK-GDCPFDFGGYFLIKGTEKVFIA   239
Sb07g00460    RREIMTETKQVSMGLLPVMVKSNLCWLH-----KLQE-SDCQFDFGGYFLIKGTEKVFIA   292
OsNRPD2b      RKDIMKETKWVTIGKLPVM-----------------E-SECEYDFGGYFLIKGMEKVFVA   296
Bd_3.1525     KRDILNETKWVSIGRLPVM-----------------K-TDCQFDYGGYFLIKGMEKAFVA   268
AtNRPD2a      KKILDVKKQDILIGSIPVMVKSILCKTSEKGKENCKK-GDCAFDQGGYFVIKGAEKVFIA   219
PtNRPD2       KNVVHTENREIIIGRIPVMVKSDLCWLT-----TVEK-GDCDFDHGGYFLIKGAEKVFIA   209
VvNRPD2       KKVIFEDNRDILIGRIPVMVKSELCWMN-----GVER-GDCEYDHGGYFLIKGAEKTFIA   234
OsNRPB2       EVTETMEYPKVFIGKVPIMLRSSYCTLFQQSEKDLTELGECPYDQGGYFIINGSEKVLIA   235
AtNRPB2       EVTETQDFTKVFIGKVPIMLRSSYCTLFQNSEKDLTELGECPYDQGGYFIINGSEKVLIA   202
                                                              Domain B
```

```
                                     380                 400                 420
NRPD2a        QEQRCLRRLWI-SDRPCWTISF-MSEIKR---------RRIYIKLVESTRSEDF-SESKI   318
Sb01g04210    QELRCLKRLWI-IDRPCWMISF-MSEMKR---------RRIYIKLVESTRSEDF-SGSKI   317
Sb06g03030    QEQRCLKRLWV-SDRPFWMISF-MHEIKR---------RRIYIKLVESTRSEDF-SGSKV   328
ZM2G128427    QELRCLRRLWI-SDRPCWTISF-MSEMKR---------RRIYIKLVESTRSEDF-SGSKI   232
```

```
OsNRPD2a     QEQKCLTRIWV-EDRPCWMVSF-LSPIRR---------RRIYIKLIDSANNEDA-SGGKI   317
Bd_9.1041    QEQRCLTRIWV-DDRPCWTVSY-MSEIKR---------KRTYVKLIDSTKSNDF-SESKI   323
Bd_6.650     QEQRCLTRIWV-ADQPCWNVSY-LSEMKR---------RRVYIKLIDSTTNNDL-NGAKI   300
Bd_2.4317    QEQRCLTRIWV-ADRPCWNVSY-LSEMKR---------RRVYIKLIDSTTNNDL-NGAKI   300
ZM2G133512   EEQRFLSRIWV-TDHPSWDASY-LSQIRR---------EKINIKLVPSKSNESC----KV   284
Sb07g00460   EEQRFLSRIWI-TDYPSWDASY-LSQIKR---------EKINIKLVPSKRNESC----KV   337
OsNRPD2b     EEQRCLSRIWI-NDSPTWEACYQRSQIRR---------EKISIKPVQSND-----GFRKV   341
Bd_3.1525    EEQRCLSRIWI-KDHPSWDASY-MSQNKR---------ERIYVKLVQSEESHGL---RKL   314
AtNRPD2a     QEQMCTKRLWI-SNSP-WTVSF-RSENKR---------NRFIVRLSENEKAEDYKRREKV   267
PtNRPD2      QEQICMKRLWI-SNSQGWTVSY-KSEVKR---------NRLIVRLVELSKLEYIKGEKKG   258
VvNRPD2      QEQICLKRLWV-SSNPTWMVAY-RPIWKR---------KRVYVKL-EPPKDENNRGGEKV   282
OsNRPB2      QEKMSTNHVYVFKKRQPNKYAY-VAEVRSMAENQNRPASSMFVRMLSRAGAKGG-SSGQY   293
AtNRPB2      QEKMSTNHVYVFKKRQPNKYAY-VGEVRSMAENQNRPPSTMFVRMLARASAKGG-SSGQY   260
                          *rmr7-1 lesion

                                     440        460        480
NRPD2a       I--TISFLYATMPVWLLFFALGISSDKEVFDMIDMQDCDASVINTISATIKESDKLCED-   375
Sb01g04210   I--SISFLYATMPVWLLFFALGISSDKEAFDVIDMQDCDASVINTISATIKESDELCEG-   374
Sb06g03030   I--SISFLYATMPVWLLFFALGISSDKEAFDVIDMQDCDASVINTISATIKESDELCKG-   385
ZM2G128427   I--SISFLYATMPVWLLFFALGISSDKEAFDVIDMQDCDASVINTISATIKESDELCGG-   289
OsNRPD2a     I--SISFLYANMPIWLMFFALGISSDKDIFDVINMEDCDACVINTITATIKESDELCEG-   374
Bd_9.1041    I--SISFLYANMPVWLMFFALGISSDKEVFDIIDFKDSDASVINMISATISESNELCEG-   380
Bd_6.650     I--SISFLYANMPIWLLFFALGVSSDKEVFDMIDMKDCDASVINAIYATIRESDELCEG-   357
Bd_2.4317    I--SISFLYANMPIWLLFFALGVSSDKEVFDMIDMKDCDASVINAISATIRESDELCEG-   357
ZM2G133512   I--NICFMGTIMPIWVAFFALGVSSDKEAFDMIDILDCDADIVNIISLTIKESHEEFEG-   341
Sb07g00460   I--TIYFMGTIIPIWVVFFALGVSSDKEAFDMIDILDCDASIVNIISSTIKESHEEFEG-   394
OsNRPD2b     I--NLYFLGATIPIWIMFFALGVSSDKEAFDIIDIQECDASMANIISATITESHEQCEG-   398
Bd_3.1525    V--RLFFLGATMPIWIMFFALGVSSDKEAFDMIDIQDCDASLVNIISATIKESDEQCEG-   371
AtNRPD2a     L--TVYFLSTEIPVWLLFFALGVSSDKEAMDLIAFDGDDASITNSLIASIHVADAVCEA-   324
PtNRPD2      L--CVYFLSTEIPLWILFFALGVRSDKEVIDLIDYASNDASIVNIFFASIHDADEKCEH-   315
VvNRPD2      L--TVYFSSTEIPIWILFFALGASSDKEVVDLIDFNIDDAGISNILVASIHEADREAEKK   340
OsNRPB2      IRATLPYIRADIPIIIVFRALGFVADKDILEHICYDFSDTQMMELLRPSLEEAFV-----   348
AtNRPB2      IRCTLPYIRTEIPIIIVFRALGFVADKDILEHICYDFADTQMMELLRPSLEEAFV-----   315

                                     500        520        540
NRPD2a       ---FRKSDKARQYVDE---LVKSSRFPPAESFDDYIARFLFP-DIS----GNRNKALFLG   424
Sb01g04210   ---FRKSDKARQYVDE---LVKSSRFPPAESFDDYIARFLFP-DIS----GNRNKALFLG   423
Sb06g03030   ---FRKSDKARQYVDE---LVKSSRFPPAESFDDYVARFLFP-DIN----GNRNKAFFLG   434
ZM2G128427   ---FRKSDKARQYVDE---LVKSSKFPPVESFDDYIAKFLFP-GIS----GNRNKALFLG   338
OsNRPD2a     ---FRKSDKARQYVDE---LIKNSKFPPAEPFDDYIAKYLFP-SIS----GNRNKALFLG   423
Bd_9.1041    ---FRKSDKARQYVDD---LVKSSKFPPAESFDDYVARFLFP-GIS----GNRNKAFFLG   429
Bd_6.650     ---FRQSDKARKYVDD---LVKSSKFPPAEPFTDYVAKYLFP-GIS----GNRNKSFFLG   406
Bd_2.4317    ---FRQSDKARKYVDD---LVKSSKFPPAEPFTDYVAKYLFP-GIS----GNRNKAWGLG   406
ZM2G133512   ---FRTPGRARQYVDE---LIRKSKFPPKESFDEYVCRYMFP-GVN----GFRSKALFLG   390
Sb07g00460   ---FRAPGRACQYVDK---LIRKSKFPPKESFDEYVCRYMFP-SVN----GVRSKALFLG   443
OsNRPD2b     ---FQREGRASEYIDK---LIRNTKFPPKGSFDEYIGRHMFP-DVS----GNRSKALFLG   447
Bd_3.1525    ---FRRGGRARQYVDE---FIKKTKFPPEQSFDGYVGRYMFPGDVS----DNRSKAFFLG   421
AtNRPD2a     ---FRCGNNALTYVEQ---QIKSTKFPPAESVDECLHLYLFP-GLQ----SLKKKARFLG   373
PtNRPD2      ---FRREDRALDYVDK---LLKKTRFPPKESIEDAISAYLFP-RLN----SRRHKARFLG   364
VvNRPD2      GMYFRRQGNAISFVDK---LVKSCKFPPGESIQECISKYLFP-NFS----GVKQKARFLG   392
OsNRPB2      ---IQNQQVALDYIGKRGATVGVTKEKRIKYAKEILQKEMLP-HVGVGEFCETKKAYYFG   404
AtNRPB2      ---IQNQLVALDYIGKRGATVGVTKEKRIKYARDILQKEMLP-HVGIGEHCETKKAYYFG   371

                                     560        580        600
NRPD2a       YMVKCLLMAFTGKRKCDNKDDFRNKRLDLPGELLGRELRAQLRLAEKRMVKAIQRDLNSD   484
Sb01g04210   YMVKCLLMAFTGKRKCDNKDDFRNKRLDLPGELLGRELRAHLRLAEKRMVKAIQRDLNSD   483
Sb06g03030   YMVKCLLMAFTGKRKCDNKDDFRNKRLDLPGELLGRELRAQLRLLERRMVKAIQRDLNND   494
ZM2G128427   YMVKCLLMAFTGKRKCDNKDDFRNKRLDLPGELLGRELRAHLRQAERRMVKAIQRDLNSD   398
OsNRPD2a     YMVKCLLMAFTGKRKCDNKDDFRNKRLDLAGELLGRELRAHIRHAERLMVKALQRDLNSE   483
Bd_9.1041    YMVKYLLMAFTGKLKCDNRDAFRNKRLELPGELLGRELRAHLRHAERLMVKAMQRDLNSD   489
Bd_6.650     YMVKCLLMAFTGKRKCDNKDDFRNKRLELPGQLLGRELRAHLRHAERLMVKAMQRDLNSD   466
Bd_2.4317    YMVKCLLKAFTGKRKCDNKDDFRNKRLELPGQLLGRELRAHLRHAERLMVKAMQRDLNSD   466
ZM2G133512   YMVKCLLMAYSGNRKCDNKDDLRNKRLDLSCQLLRRELWTHIKRAERRMVKLMQRDLSND   450
Sb07g00460   YMVKCLLMAYSGNRKCDNKDDFRNKRLGLACQLLRRELWTHIKRAEWRMVKLMQRDLSND   503
```

```
OsNRPD2b    YMVRCLLLASSGNRKSDNRDDFRNKRLDLACELLQRELWVHIMHAQKRMVKVMQRHLSGD   507
Bd_3.1525   YMVKCLLMAYSGHRKCDDRANFRNKRLDLACQLLRRELWVHLRHAQRRMVKIMQRHLSGD   481
AtNRPD2a    YMVKCLLNSYAGKRKCENRDSFRNKRIELAGELLEREIRVHLAHARRKMTRAMQKHLSGD   433
PtNRPD2     YMVKCLLEAYTGHRKCDNRDSFRNKRFELASELLERELKVHVSHALRRMTKALQRDLYGD   424
VvNRPD2     YMVKCLLQAYTGRRKCDNRDDFRNKRLELAGELLERELRVHIRHAERRMVKAMQRELYGD   452
OsNRPB2     YIIHRLLMCALGRRAEDDRDHYGNKRLDLAGPLLGGLFRMLFRKLTRDVRSYVQKCVDNG   464
AtNRPB2     YIIHRLLLCALGRRPEDDRDHYGNKRLDLAGPLLGGLFRMLFRKLTRDVRSYVQKCVDNG   431
                              Domain C

                                    620                 640                 660
NRPD2a      RELQDLERYIDASIVTNGLNRAFSTGSWCHPYKRAERCSGIVATLRRTNPLQMMSDLRKT   544
Sb01g04210  RELQDLERYIDASIVTNGLSRAFSTGSWCHPYKRAERCSGIVATLRRTNPLQMMSDLRKT   543
Sb06g03030  RELQDLERYIDASIVTNGLNRAFSTGSWCHPYKRAERCSGIVATLRRTNPLQMMSDLRKT   554
ZM2G128427  RELQDLERYIDASIVTNGLNRAFSTGSWCHPYKRAERCSGIVATLRRTNPLQMMSDLRKT   458
OsNRPD2a    RELQEFDHYLDASIITNGLNRAFSTGSWCHPYKRNERCAGIVATLRRTNPLQMISDLRKT   543
Bd_9.1041   RDLQFPLGYLDPTIITNGINRAFATGSWCHPYKRNERCSGVVATLRRTNPLQMMSDLRKS   549
Bd_6.650    RDLQFPLRYLDASIITNGINRAFATGSWSHPYIRNERCSGIVATLRRTNPLQMMSDLRKS   526
Bd_2.4317   RDLQFPLRYVDASIITNGINRAFATGSWSHPYIRNERCSGIVATLRRTNPLQMMSDLRKS   526
ZM2G133512  GNLQDLRRYVDASIITNGLNRAFSTGSWRHPYKK-ERCSGVVATLRRTNPLQMMSDVRKT   509
Sb07g00460  GNLQDLRRYVDASIITNGLNRAFSTGSWRHPYLK-ARCSGVVATLRRTNPLQMMSDLRKT   562
OsNRPD2b    GDLQPLECYVHASIVTNGLNRAFSTGSWCHPFNKRERCSGIVATLRRTNPLQMMSDMRKT   567
Bd_3.1525   GDLQVLDHYVDTSIVTNGLNRAFSTGSWCHPYK-YERCSGIVGNLRRTNPLQMMSDLRKT   540
AtNRPD2a    GDLKPIEHYLDASVITNGLSRAFSTGAWSHPFRKMERVSGVVANLGRANPLQTLIDLRRT   493
PtNRPD2     RDVHPIEHYLDASIVTNGLTRAFSTGAWCHPFKWMERVSGVVGNLGRANPLQTMIDLRKT   484
VvNRPD2     RDLRPIENYLDASIITNGLSRAFSTGQWSHPFKRMERISGVVATLRRTNPLQMTADMRKT   512
OsNRPB2     KEV-NLQFAIKAKTITSGLKYSLATGNWGQANQAGTR-AGVSQVLNRLTYASTLSHLRRL   522
AtNRPB2     KEV-NLQFAIKAKTITSGLKYSLATGNWGQANAAGTR-AGVSQVLNRLTYASTLSHLRRL   489

                                    680                 700                 720
NRPD2a      RQRVAYAGKAGDARYPNPSYWGKLCFMSTPDGENCGLVKNLAVTAIVS-SRVMQPLIESF   603
Sb01g04210  RQRVAYAGKAGDARYPNPSYWGKLCFMSTPDGENCGLVKNLAVTAIVS-SRVVQPLIESF   602
Sb06g03030  RQRVAYAGKAGDARYPNPSYWGKLCFLSTPDGENCGLVKNLAVTAIVS-SRVGQPLIESF   613
ZM2G128427  RQRVAYAGKAGDARYPNPSYWGKLCFMSTPDGENCGLVKNLAVTSIVS-SKVVQPLIESF   517
OsNRPD2a    RQRVAYAGKAGDARYPNPSYWGKLCFMSTPDGENCGLVKNLAVTATVS-SRVAPPLIDRF   602
Bd_9.1041   RQQVAYAGKAGDARYPNPSYWGKMCFMSTPDGENCGLVKNLAVTAIVS-SRVVQPLIDRF   608
Bd_6.650    RQQVAYAGKVGDARYPNPSYWGKMCFMSTPDGENCGLVKNLAVTAIVS-SRVVQPLIDRF   585
Bd_2.4317   RQQVAYAGKVGDARYPNPSYWGKMCFMSTPDGENCGLVKNLAVTAIVS-SRVVQPLIDRF   585
ZM2G133512  RQWFAYAGTAGDARYPNPSYWGKLCFLSTPDAEKCGFVKNLAVTAVVS-SVVRKPLIDTF   568
Sb07g00460  RQWVAYSGAVGDARYPNPSFWGKLCFLSTPDGEKCGFVKNLAVTAVVS-SVVRKPLIDTF   621
OsNRPD2b    RQWVAYAGKAGDARYPNPSYWGKLCFLSTPDGEKCGFVKNLAITAIVS-CLAREPSVDAL   626
Bd_3.1525   RQLSAYFGNAGDARYPNPSYWGKLCFLSTPDGEKCGFVKNLAVTAVVS-SVMRKPLMDLF   599
AtNRPD2a    RQQVLYTGKVGDARYPHPSHWGRVCFLSTPDGENCGLVKNMSLLGLVS-TQSLESVVEKL   552
PtNRPD2     RQQVLYTGKVGDARYPHPSHWGRVCFLSTPDGENCGLVKNLAVTGVVS-TNISESLVDKL   543
VvNRPD2     RQQVQYTGKVGDARYPHPSHWGKVCFLSTPDGENCGLVKNLAITGLVS-TEVLDPLVDKL   571
OsNRPB2     NSPIGREGKLAKPRQLHNSHWGMMCPAETPEGQACGLVKNLALMVYITVGSAANPILEFL   582
AtNRPB2     NSPIGREGKLAKPRQLHNSQWGMMCPAETPEGQACGLVKNLALMVYITVGSAAYPILEFL   549
                                Domain D
                           *rmr7-2 lesion

                                    740                 760                 780
NRPD2a      ISCGMSKLNDIPTEHIQRMDKIFLNGNWVGSCENSASFVFRLRCMRRSSLIDPQ------   657
Sb01g04210  ISCGMSKLNDIPTEHIQRMDKIFLNGNWVGSCKDSASFVFRLRCMRRSSLIDPQ------   656
Sb06g03030  ISCGMSKLNDIPTEHIQRMDKIFLNGNWVGSCKDSASFVFRLRCMRRSSMIDPQ------   667
ZM2G128427  ISCGMNKLNDIPTEHIQRMDKIFLNGNWLGSCSDSASFVFRLRCMRRSSLIDPQ------   571
OsNRPD2a    ISCGMNKLHEIPTEEVPRMDKIFLNGDWVGSCSDPASFVLRLRCMRRSGLIDPQ------   656
Bd_9.1041   VSCGMNKLDEIPAGQIPKMDKIFLNGNWVGSCTDPASFVMRLRCMRRGNLIDPQ------   662
Bd_6.650    VSCGMNKLDEISAKEIPKMDKIFLNGDWIGSCTDPASFVMRLRCMRRANLIDPQ------   639
Bd_2.4317   VSCGMNKLDEISAKEIPKMDKIFLNGDWIGSCTDPASFVMRLRCMRRANLIDPQ------   639
ZM2G133512  VSCGMKKLDDISLQDISGKDRIFLNGSLLGVCADPHELTLRLRSLRRSKLIDPQ------   622
Sb07g00460  VSCGMKKLDDISLQDISGKDRIFLNGSLLGVCADPHELTLRLRSLRRSKLIDPQ------   675
OsNRPD2b    VSCGMKKLDELLQEISGKDRIFLNGNLVGVCADSVEFVLHLRSMRRRKQIDAQ------   680
Bd_3.1525   VSCGMKKLNEVRVQELHGTDKTFLNGNLIGVCANPGEFVTHLRNMRRSNKIDRQKYVLKR   659
AtNRPD2a    FACGMEELMDDTCTPIFGKHKVLLNGDWVGLCADSESFVAELKSRRRQSELPRE------   606
PtNRPD2     FDSGMEKLVDDTYTKLDGKHKVFLNGEWVGVCEDSCLFVGELRSMRRRRELPYQ------   597
```

```
VvNRPD2      FDCGMEKLVDDTSTKLSGKNKVFLDGDWVGVCEDPISFVVELRTKRRHKELPQQ------   625
OsNRPB2      EEWGTENFEEISPAVIPQAAKIFVNGCWVGIHRNPDLLVKTLRRLRRQIDVNTE------   636
AtNRPB2      EEWGTENFEEISPSVIPQATKIFVNGMWVGVHRDPDMLVKTLRRLRRRVDVNTE------   603

                                  800                 820                 840
NRPD2a       --------------VEIKRDKHH--NEVRVFTDAGRILRPLLVVEN----------LNKIR   692
Sb01g04210   --------------VEIKRDKHH--KEVRVFSDAGRILRPLLVVEN----------LKKIR   691
Sb06g03030   --------------VEIKRDKHH--KEVRVFSDAGRILRPLLVVEN----------LNKIR   702
ZM2G128427   --------------VEIKRDKHY--KEVRLFSDAGRILRPLLVVEN----------LNKIR   606
OsNRPD2a     --------------VEIKRDKHQ--REVRVFSDAGRILRPLLVVEN----------LNKIR   691
Bd_9.1041    --------------VEIKRDKHQIPGEVRVFSDAGRILRPLLVVEN----------LNKIR   699
Bd_6.650     --------------VEIKRDKHQFPGEVRVFSDAGRILRPLLVVEN----------LNKIR   676
Bd_2.4317    --------------VEIKRDKHQFPGEVRVFSDAGRILRPLLVVEN----------LNKIR   676
ZM2G133512   --------------VEIKRDKHH--KEVRVFSDPGRIMRPLLVVEN----------LRRIT   657
Sb07g00460   --------------VEIKRDKHH--KEVRVLCDPGRILRPLLVVEN----------LRRIT   710
OsNRPD2b     --------------VEIKRDKQN--KEVRIFSDPGRILRPLLIVEN----------LRNIM   715
Bd_3.1525    TELSSCGQPSEDFDVEIKRDMQH--KEVRVFSDAGRILRPLLIVEN----------LKSMT   708
AtNRPD2a     --------------MEIKRDKDD--NEVRIFTDAGRLLRPLLVVEN----------LQKLK   641
PtNRPD2      --------------VEIKRDEQQ--REVRIFSDAGRILRPLLVVEN----------LDKIK   632
VvNRPD2      --------------VEIKRDEQQ--GEVRIFSDAGRILRPLLVVEN----------LKKVK   660
OsNRPB2      --------------VGVVRDIRL--KELRLYTDYGRCSRPLFIVENQRLLIKKRHIRALQ   680
AtNRPB2      --------------VGVVRDIRL--KELRIYTDYGRCSRPLFIVDNQKLLIKKRDIYALQ   647

                                  860                 880                 900
NRPD2a       --KPKGRSF-SFHELMQQEIIEFIGVEEEEDIQCAWGIRHLFESE---------------   734
Sb01g04210   --KPKGRSF-SFQELMQQEIIEFIGVEEEEDIQCAWGIRHLFESE---------------   733
Sb06g03030   --KPKGRSF-SFQELMQQEIIEFIGVEEEEDIQCAWGIRHLFESE---------------   744
ZM2G128427   --KPKGRSF-SFQELMQQEIIEFIGVEEEEDIQCAWGIRHLFESE---------------   648
OsNRPD2a     --RPKGSSY-SFQWLMQQEIIEFIGVEEEEDIRSAWGIRNLFESEEEAPMVKMNKAEDVF   748
Bd_9.1041    --KPKDGSY-SFQALMQQEIIEYIGVEEEEDILCAWGIRHLFPGS---------------   741
Bd_6.650     --KSKDRPY-TFQALLQQEIIEYIGVEEEEDIQCAWGIRHLFPSS---------------   718
Bd_2.4317    --KSKDRHY-TFQALLQQEIIEYIGVEEEEDIQCAWGIRHLFPSS---------------   718
ZM2G133512   --RPKDGLY-SFQELIDQNIVELIGVEEEEDIQCAYGIRHLFSSRE--------------   700
Sb07g00460   --RPKDGLY-SFQELIDQNIVELIGVEEEEDIQCASGIRHLFSGEK--------------   753
OsNRPD2b     --NRKNGSY-SFQELMDQNIIELIGVEEEEDIRCAYGIRHLFAGDE--------------   758
Bd_3.1525    TIKQKNGSY-SFQELVDKNIIELIGVEEEEDIRCACAIRDLFSGDN--------------   753
AtNRPD2a     --QEKPSQY-PFDHLLDHGILELIGIEEEEDCNTAWGIKQLLK-----------------   681
PtNRPD2      --AFKGGNY-IFTSLLDKGIIEFIGTEEEEDCCTAWGIKFLLADIE--------------   675
VvNRPD2      --TFKGDDF-TFQSLLDKGIVELIGAEEEEDCSTAWGIKYLLKGH---------------   702
OsNRPB2      --QRETPEE-GWHDLVAKGFIEYIDTEEEETTMISMTINDLIGARH--------------   723
AtNRPB2      --QRESAEEDGWHHLVAKGFIEYIDTEEEETTMISMTISDLVQARL--------------   691

                                  920                 940                 960
NRPD2a       -------GAISSYTHCELDPSFLLGLSCGIIPFANHNFARRVLYQSEKHSQQAIGYSTTN   787
Sb01g04210   -------GAISSYTHCELDPSFLLGLSCGIIPFANHNFARRVLYQSEKHSQQAIGYSTTN   786
Sb06g03030   -------GAISSYTHCELDPSFLMGLSCGIIPFANHNFARRVLYQSEKHSQQAIGYSTTN   797
ZM2G128427   -------GAISSYTHCELDPSFILGLSCGIIPFANHNFARRVLYQSEKHSQQAIGYSTSN   701
OsNRPD2a     NVKRKIGGEVSGYTHCELDLSFLLGLSCGIIPFANHNFARRVLYQSEKHSQQAIGYSTTN   808
Bd_9.1041    ------GEDFSGYTHCELDLSFLLGLSCSLIPFANHNFARRVLYQSEKHSQQAIGYSTTN   795
Bd_6.650     ------GEKVSGYTHCELDLSFLLGLSCGLIPFANHNFARRVLYQSEKHSQQAIGYSTTN   772
Bd_2.4317    ------GEKVSGYTHCELDLSFLLGLSCGLIPFANHNFARRVLYQSEKHSQQAIGYSTTN   772
ZM2G133512   ----KEDWSSSGYTHCELDPSFLLGLSCSLIPFGNHDNARRVQMQAEKISQQAIGYSPTN   756
Sb07g00460   ------EDRSSGYTHCELDPSFLLGLSCSLIPFANHDNGKRVLMQAEKISQQAIGYSPTN   807
OsNRPD2b     ------EKNFSFYTHCELDPSFLLGLSCSIIPFANHDTAKRILMQAEKISQQAIGYSTTN   812
Bd_3.1525    ------EEGFLYYTHCELDPSFLLGLSCGIIPFANHNNARRVLMQAEKLSQQAIGYSSTN   807
AtNRPD2a     ---------EPKIYTHCELDLSFLLGVSCAVVPFANHDHGRRVLYQSQKHCQQAIGFSSTN   733
PtNRPD2      ------GKQPMKYSHCELDMSFLLGLSCGIIPFANHDHARRVLYQAQKHSQQAIGFSTTN   729
VvNRPD2      ------DDPPVKYTHCELDMSFLLGLSCGIIPYANHDHARRVLYQSEKHSQQAIGFSTTN   756
OsNRPB2      ---NPEEAYSETYTHCEIHPSLILGVCASIIPFPDHNQSPRNTYQS-AMGKQAMGIYVTN   779
AtNRPB2      ---RPEEAYTENYTHCEIHPSLILGVCASIIPFPDHNQSPRNTYQS-AMGKQAMGIYVTN   747

                                  Domain E
```

```
                                   980                 1000                1020
NRPD2a       PHIRVDTLSHQLYYPQRPLFKTVIADCLGRS--DYASFGRKNDFARPE---YFNGQNAIV     842
Sb01g04210   PHIRVDTLSHQLYYPQRPLFKTVIADCLGRS--DYTTFGRKDDFMRPE---YFNGQNAIV     841
Sb06g03030   PHIRVDTLSHQLYYPQRPLFKTVIADCLGRS--DYTAFGRKDDYTRPE---YFNGQNAIV     852
ZM2G128427   PRIRVDTLSHQLYYPQRPLFKTVIADCLG----------------RPE---YFNGQNAIV     742
OsNRPD2a     PHIRVDTLSHQLYYPQRPLFKTVIADCIGRS--EY-TFGRKDDFARPE---YFNGQNAIV     862
Bd_9.1041    QLTRVDTLSHQLYYPQRPLFKTVTADCIGRS--DY-TIGRTDDFARPE---YFNGQNAIV     849
Bd_6.650     PLTRVDTHSHQLYYPQRPLFKTVTADCIGRS--DY-TIGRKDDFARPE---YFNGQNAIV     826
Bd_2.4317    PLTRVDTHSHQLYYPQRPLFKTVTADCIGRS--DY-TIGRKDDFARPE---YFNGQNAIV     826
ZM2G133512   SQYRLDTLSHQMFYPQRPLFRTVVSYGLGEAKTDC-SSGRKDDFNTPE---YFNGQNAIV     812
Sb07g00460   SHTRLDTLSHQIFYPQRPLFKTVVSYGLGKAETAY-SFGRKDDFNTPE---YFNGQNAIV     863
OsNRPD2b     PLFRVDTHSHQLYYPQRPLFKTVAADCLGKR--DY-TSGSKHDFARPE---YFNGQNAIV     866
Bd_3.1525    SQYRVDTLFHQMYYPQKPLFKTVVADCIGKS--DH-NFGEEDDFTRPENFPYFNGQNAIV     864
AtNRPD2a     PNIRCDTLSQQLFYPQKPLFKTLASECLKKE----------------V---LFNGQNAIV     774
PtNRPD2      PNIRVDTLSHQLHYPQRPLFRTMISDCL--------------VLPKPE---LFNGQNAIV     772
VvNRPD2      PNIRVDTLSHQLYYPQRPLFRTMISDCLGKP--GY-SEGHKGIVPRPE---YFNGQIAIV     810
OsNRPB2      YQLRMDTLAYVLYYPQKPLVTTRAMEHLHFR-----------------Q---LPAGINAIV     820
AtNRPB2      YQFRMDTLAYVLYYPQKPLVTTRAMEHLHFR-----------------Q---LPAGINAIV     788
```

```
                                  1040                1060                1080
NRPD2a       AVNVHQGFNQEDSLVMNRASLERGMFRTEHLRSYKA VENK---DG-TKRLKLKEKIDFG     898
Sb01g04210   AVNVHQGFNQEDSLVMNRASLERGMFRTEHLRSYKADVENK---DG-TKRLKLKEKIDFG     897
Sb06g03030   AVNVHQGFNQEDSLVMNRASLERGMFRTEHLRSYKTDVENK---DG-TKRLKLKEKIDFG     908
ZM2G128427   AVNVHQGFNQEDSLVMNRASLERGMFRTEHLRSYKADVENK---DG-TKRLKLKEKIDFG     798
OsNRPD2a     AVNVHQGFNQEDSVVMNRASLERGMFRTEHFRNYKAEVENKGGPGG-NKRLKMKDKIDFG     921
Bd_9.1041    AVNVHQGFNQEDSLVMNRASLERGMFRTELIRSYKADVETK---EP-AKRLKLKEKVDFG     905
Bd_6.650     AVNVHQGFNQEDSLVMNRASLERGMFRTEHIRSYKAEVETK---EP-TKRLKLKEKVDFG     882
Bd_2.4317    AVNVHQGFNQEDSLVMNRASLERGMFRTEHIRSYKAEVETK---EP-IKRLKLKEKVDFG     882
ZM2G133512   SVNVHQGFNQEDSLVMNRASLERGMFRTLHFKSYKAQVENK---EI-TRRLKHRENINFG     868
Sb07g00460   SINVHQGFNQEDSLVLNRASLERGMFRTLHLKSYKAQVENK---EI-TRRLKHRESINFG     919
OsNRPD2b     SISVHQGFNQEDSLVLNRASLERGMFRTQHFKSYKALIENK---EI-TKRLKHKENINFG     922
Bd_3.1525    SISVHQGFNQEDSLVFNRGSLERGMFRTQHFKSYKTQIENK---EV-TRRLKYREKIDFG     920
AtNRPD2a     AVNVHLGYNQEDSIVMNKASLERGMFRSEQIRSYKAEVDAK---DS-EKRKKMDELVQFG     830
PtNRPD2      AVNVHLGYNQEDSLVMNRASLERGMFRSEHIRSYKAEVDNK---ELTDKRRKSEDSITFG     829
VvNRPD2      AVNVHLGYNQEDSLVMNRASLERGMFRSEHIRSYKSEVDNN---ESLDKKRKSEDSVHFG     867
OsNRPB2      AIACYSGYNQEDSVIMNQSSIDRGFFRSLFFRSYRDE----------EKKMGTLVKEEFG     870
AtNRPB2      AISCYSGYNQEDSVIMNQSSIDRGFFRSLFFRSYRDE----------EKKMGTLVKEDFG     838
```

Domain F

```
                                  1100                1120                1140
NRPD2a       K------TESKRGRVDNLDDDGLPYVGASLQTNDIVIGKVSE--------------SGEDH     939
Sb01g04210   K------TESKRGRVDNLDDDGLPYIGASLQTNDIVIGKVSE--------------SGEDH     938
Sb06g03030   K------TESKRGRVDNLDDDGLPYIGASLQTNDIVIGKVSE--------------SGEDH     949
ZM2G128427   K------TESKRGRVDNLDDDGLPYIGASLQTNDIVIGKVSE--------------SGEDH     839
OsNRPD2a     K------MQSKRGRVDNLDDDGLPYVGASLQSGDIVIGKVSE--------------SGEDH     962
Bd_9.1041    K------MQSKRGRVDSLDDDGLPYVGASLQSGDIVIGKVSE--------------SGEDH     946
Bd_6.650     K------MQSKRGRVDNLDDDGLPYVGASLQSGDIVIGKVSE--------------SGEDH     923
Bd_2.4317    K------MQSKRGRVDNLDDDGLPYVGASLQSGDIVIGKVSE--------------SGEDH     923
ZM2G133512   K------VQSKRGKVDSLDSDGLPYVGASLQSGDIVIGKVTE--------------SGEDH     909
Sb07g00460   K------VQSKRGKVDSLDSEGLPYVGASLQSGDIVIGKVSE--------------SGEDH     960
OsNRPD2b     K------TPSKKGLVDSLDIDGLPYIGASLQSNDIIIGKVSD--------------SGEDH     963
Bd_3.1525    K------TQSKRGRVDSLDIDGLPYIGASLQSGDIVIGKVSE--------------SGEDH     961
AtNRPD2a     K------THSKIGKVDSLEDDGFPFIGANMSTGDIVIGRCTE--------------SGADH     871
PtNRPD2      K------IQSKIGRVDSLDDDGFPFIGANMQSGDIVIGKCAE--------------SGADH     870
VvNRPD2      K------MQSKIGRVDSLDDDGFPFIGANLQNGDIVIGRCAE--------------SGVDH     908
OsNRPB2      RPNRENTMGMRHGSYDKLDDDGLAPPGTRVSGEDVIIGKTSPIPQDDAQGQATRYTKRDH     930
AtNRPB2      RPDRGSTMGMRHGSYDKLDDDGLAPPGTRVSGEDVIIGKTTPISQDEAQGQSSRYTRRDH     898
```

Domain G

```
                                  1160                1180                1200
NRPD2a       SIKLKHTEKGMVQKVLLSANDEGKNFAVVTLRQ---------------------------     972
Sb01g04210   SIKLKHTEKGMVQKVLLSANDEGKNFAVVTLRQ---------------------------     971
Sb06g03030   SIKLKHTEKGMVQKVLLSANDEGKNFAVVTLRQ---------------------------     982
```

```
ZM2G128427   SIKLKHTEKGMVQKVLLSANDEGKNFAVVTLRQ---------------------------    872
OsNRPD2a     SIKLKHTEKGMVQRVLLSANDEGKNFAVVTLRQ---------------------------    995
Bd_9.1041    SIKLKHTEKGMVQRVLLSANDEEKNFAVVTLRQ---------------------------    979
Bd_6.650     SIKMKHTEKGMAQRVLLSANDEGKNFAVVTLRQ---------------------------    956
Bd_2.4317    SIKMKHTEKGMVQRVLLSANDEGKNFAVVTLRQ---------------------------    956
ZM2G133512   SAKLMHTEKGMVDKVVLSANDDGVNFATVTLRQ---------------------------    942
Sb07g00460   SAKLMHTEKGMVDKVVLSANDDGVNFATVTLRQ---------------------------    993
OsNRPD2b     SIKLLHTEKGIVEKVVLSATDDGTNSAFVTLRQ---------------------------    996
Bd_3.1525    SMKLMHTEKGMVEKVVLSANDDGKNSAVVTLRQKCHLVLPVPDTAQRPPEMVISLSRTGN   1021
AtNRPD2a     SIKLKHTERGIVQKVVLSSNDEGKNFAAVSLRQ---------------------------    904
PtNRPD2      SVKLKHTERGMVQKVVLSSNDEGKNFAVVSLRQ---------------------------    903
VvNRPD2      SIKLKHTERGMVQKVVVSANDDGKNFAVVSLRQ---------------------------    941
OsNRPB2      STSLRHSESGMVDQVLLTTNADGLRFVKVRMRS---------------------------    963
AtNRPB2      SISLRHSETGMVDQVLLTTNADGLRFVKVRVRS---------------------------    931

                                 1220                1240                1260
NRPD2a       ---------------------VRTPCLGDKFSSMHGQKGVVGFLESQENFPFTHEGIVPD   1011
Sb01g04210   ---------------------VRTPCLGDKFSSMHGQKGVVGFLESQENFPFTHKGIVPD   1010
Sb06g03030   ---------------------VRTPCLGDKFSSMHGQKGVVGFLESQENFPFTHEGIVPD   1021
ZM2G128427   ---------------------VRTPCLGDKFSSMHGQKGVVGFLESQENFPFTHDGIVPD    911
OsNRPD2a     ---------------------VRSPCLGDKFSSMHGQKGVVGFLESQENFPFTYQGIVPD   1034
Bd_9.1041    ---------------------VRSPCVGDKFSSMHGQKGVIGFLESQENFPFTCQGIVPD   1018
Bd_6.650     ---------------------VRSPCVGDKFSSMHGQKGVIGFLESQENFPFTCQGIVPD    995
Bd_2.4317    ---------------------VRSPCVGDKFSSMHGQKGVIGFLESQENFPFTCQGIVPD    995
ZM2G133512   ---------------------SRSPCVGDKFASMHGQKGVVGLLDSQENFPFTSQGIVPD    981
Sb07g00460   ---------------------SRSPCVGDKFASMHGQKGVVGLLDSQENFPFTCQGIVPD   1032
OsNRPD2b     ---------------------TRSPRIGDKFASMHGQKGVIGFLDSQENFPFTHQGIVPD   1035
Bd_3.1525    FSSDKKWRVLHINEELQQTTKVRSPCVGDKFASMHGQKGVVGLLDSQENFPFTFQGIVPD   1081
AtNRPD2a     ---------------------VRSPCLGDKFSSMHGQKGVLGYLEEQQNFPFTIQGIVPD    943
PtNRPD2      ---------------------VRSPCLGDKFSSMHGQKGVLGFLESQENFPFTIQGVVPD    942
VvNRPD2      ---------------------VRTPCLGDKFSSMHGQKGVLGFLESQENFPFTIQGIVPD    980
OsNRPB2      ---------------------VRIPQIGDKFSSRHGQKGTVGMTYTQEDMPWTIEGITPD   1002
AtNRPB2      ---------------------VRIPQIGDKFSSRHGQKGTVGMTYTQEDMPWTIEGVTPD    970
                                         Domain H

                                 1280                1300                1320
NRPD2a       IVINPHAFPTRQTPGQLLEAALGKGIAC-----------KGTMRYATPFTTASVDVIAEQ   1060
Sb01g04210   IVINPHAFPTRQTPGQLLEAALGKGIAC-----------KGTMRYATPFTTASVDVIAEQ   1059
Sb06g03030   IVINPHAFPTRQTPGQLLEAALGKGIAC-----------KGTMRYATPFTTASVDVIAEQ   1070
ZM2G128427   IVINPHAFPTRQTPGQLLEAALGKGIAC-----------KGTMRYATPFTTASVDVIAEQ    960
OsNRPD2a     IVINPHAFPTRQTPGQLLEAALGKGIAL-----------GGTMRYATPFTTASFDVITDQ   1083
Bd_9.1041    IVINPHAFPTRQTPGQLLEAALGKGIAL-----------GSAMRYATPFTTASLEVISEQ   1067
Bd_6.650     VVINPHAFPTRQTPGQLLEAALGKGIAL-----------GGAMRYATPFTPASLEVISEQ   1044
Bd_2.4317    VVINPHAFPTRQTPGQLLEAALGKGIAL-----------GGAMRYATPFTPASLEVISEQ   1044
ZM2G133512   MVINPHGFPTRQTPGQLLEAALGKGIAL-----------GGKVRYATPFTTPTVEVIAEQ   1030
Sb07g00460   IVINPHGFPTRQTPGQLLEAALGKGIAM-----------GGKVRYATPFTTPTVDVITEQ   1081
OsNRPD2b     IVINPHGFPTRQTPGQLLEAALGKGIAL-----------GGATRYATPFTSPSVEVITEQ   1084
Bd_3.1525    IVINPHGFPTRQTPGQLLEAALGKGIAL-----------GGMTRYATPFTTPSVDVITEQ   1130
AtNRPD2a     IVINPHAFPSRQTPGQLLEAALSKGIACPIQKEGSSAAYTKLTRHATPFSTPGVTEITEQ   1003
PtNRPD2      IVINPHAFPSRQTPGQLLEAALGKGIAC-----------GGSKRYATPFSTLSVDDIIDQ    991
VvNRPD2      IVINPHAFPSRQTPGQLLEAALGKGIAC-----------GGLLRHATPFSTLSVDAIADQ   1029
OsNRPB2      IIVNPHAIPSRMTIGQLIECIMGKVAAH-----------MGKEGDATPFTDVTVDNISKA   1051
AtNRPB2      IIVNPHAIPSRMTIGQLIECIMGKVAAH-----------MGKEGDATPFTDVTVDNISKA   1019
                  Domain H

                                 1340                1360                1380
NRPD2a       LHR-------------------AGYSRWGSENVLNGRTGERVQSLVFMGPTFYQRLIHMS   1101
Sb01g04210   LHR-------------------AGYSRWGTENVLNGRTGERMQSLIFMGPTFYQRLIHMS   1100
Sb06g03030   LHK-------------------AGYSRWGAENVLNGRTGERMQSLVFMGPTFYQRLIHMS   1111
ZM2G128427   LHK-------------------AGYSRWGAENVLNGRTGERMKSLVFMGPTFYQRLIHMS   1001
OsNRPD2a     LHK-------------------AGFSRWGAESVLNGRTGERMHSLIFMGPTFYQRLIHMA   1124
Bd_9.1041    LHK-------------------AGFSGGGTESVLNGQTGERMHSLIFMGPNFYQRLTHMA   1108
Bd_6.650     LHKFYVIAIAKYVYVSMHALTWAGFSRSGAESVINGRTGERMHSLIFMGPNFYQRLTHMA   1104
Bd_2.4317    LHK-------------------AGFSRGGAESVINGRTGERMHSLIFMGPNFYQRLTHMA   1085
```

```
ZM2G133512   LHK-------------------AGFCRWGEESVLNGQTGERMKSLVFMGPTFYQRLVHMA   1071
Sb07g00460   LHK-------------------AGFSRWGEESVLNGQTGERMKSLVFMGPTFYQRLVHMA   1122
OsNRPD2b     LHK-------------------AGFSRWGGESVINGRTGERAASPVFTGPTFYQRLHHMA   1125
Bd_3.1525    LHK-------------------AGFSRWGGESVLNGQNGERMQSLVFMGPAFYQRLHHMA   1171
AtNRPD2a     LHR-------------------AGFSRWGNERVYNGRSGEMMRSMIFMGPTFYQRLVHMS   1044
PtNRPD2      LHR-------------------AKFSRWGNERVYNGRTGEMVRSLIFMGPTFYQRLVHMA   1032
VvNRPD2      LHR-------------------AGFSRWGHERVYNGRTGEMLRSLIFMGPTFYQRLIHMA   1070
OsNRPB2      LHK-------------------CGYQMRGFETMYNGHTGRKLTAMIFLGPTYYQRLKHMV   1092
AtNRPB2      LHK-------------------CGYQMRGFERMYNGHTGRPLTAMIFLGPTYYQRLKHMV   1060
             Domain I
```

rmr7-3 lesion*

```
                             1400                1420                1440
NRPD2a       EDKVKFRNTGPVHPLTRQPVADRKRFGGVKFGEMERDCLLAHGSAANLHERLFLLSDFSQ   1161
Sb01g04210   EDKVKFRNTGPVHPLTRQPVADRKRFGGVKFGEMERDCLLAHGSAANLHERLFMLSDFSQ   1160
Sb06g03030   EDKVKFRNTGPVHPLTRQPVADRKRFGGVKFGEMERDCLLAHGSAANLHERLFMLSDFSQ   1171
ZM2G128427   EDKVKYRNTGPVHPLTRQPVADRKRFGGVKFGEMERDCLLAHGSAANLHERLFMLSDFSQ   1061
OsNRPD2a     EDKVKFRNTGPVHPLTRQPVADRKRFGGVKFGEMERDCLLAHGAAANLHERLFMLSDFSQ   1184
Bd_9.1041    EDKVKFRNTGPVHPLTRQPVADRKRFGGVKFGEMERDCLLAHGAAANLHERLFMLSDFAQ   1168
Bd_6.650     EDKVKFRNTGPVHPLTRQPVADRKRFGGVKFGEMERDCLLAHGAAANLHERLFMLSDFSQ   1164
Bd_2.4317    EDKVKFRNTGPVHPLTRQPVADRKRFGGVKFGEMERDCLLAHGAAANLHERLFMLSDFSQ   1145
ZM2G133512   EDKVKFRNTGPVHPLTRQPVEDKKRFGGVKFGEMERDCMLAHGAAANLHERLFTLSDFSR   1131
Sb07g00460   EDKVKFRNTGPVHPVTRQPVEDKKRFGGVKFGEMERDCMLAHGAAANLHERLFTLSDFSQ   1182
OsNRPD2b     EDKVKFRNTGPVHPLTRQPVEDRRRYGGVKFGEMERDCLLAHGAAANLHERLFLLSDVSR   1185
Bd_3.1525    VDKVKLRNTGPVHPLTRQPVEDKKRFGGVKFGEMERDCLLAHGATANVHERLFRVSDLSE   1231
AtNRPD2a     EDKVKFRNTGPVHPLTRQPVADRKRFGGIKFGEMERDCLIAHGASANLHERLFTLSDSSQ   1104
PtNRPD2      EDKVKFRNTGPVHPLTRQPVADRKRFGGIKFGEMERDCLIAHGASANLHERLFTLSDSSE   1092
VvNRPD2      EDKVKFRNTGPVHPLTRQPVSDRKRFGGIKFGEMERDCLIAHGAAANLHERLFTLSDSAY   1130
OsNRPB2      DDKIHSRGRGPVQILTRQPAEGRSRDGGLRFGEMERDCMIAHGAAFFLKERLFDQSDAYR   1152
AtNRPB2      DDKIHSRGRGPVQILTRQPAEGRSRDGGLRFGEMERDCMIAHGAAHFLKERLFDQSDAYR   1120
             Domain I
```

```
                             1460                1480                1500
NRPD2a       MHICQTCERVANVVMRSVP----GGKKIRGPYCGFCKSSENIVRINVPYGAKLLYQELFS   1217
Sb01g04210   MHICQTCERVANVVMRSVS----GGKKIRGPYCGFCKSSENIVRINVPYGAKLLYQELFS   1216
Sb06g03030   MHICQTCERVANVVMRSVP----GGKKIRGPYCGFCKSSENIVRINVPYGAKLLYQELFS   1227
ZM2G128427   MHICQTCERVANVVMRSVP----GGKKIRGPYCGFCKSSENIVRINVPYGAKLLYQELFS   1117
OsNRPD2a     MHVCQTCERVANVIMRPVP----GGKKIRGPYCGFCRSSENIVRINVPYGAKLLYQELFS   1240
Bd_9.1041    MHICQTCQRAANVVMRAIP----GGKKIRGPYCGFCRSSENKVRIAVPYGAKLLYQELFS   1224
Bd_6.650     MHICRTCERVANVVMRSVP----GGKRIRGPYCGFCRSSENTVRIAVPYGAKLLYQELFC   1220
Bd_2.4317    MHICRTCERVANVVMRGVP----GGKRIRGPYCGFCRSSENTVRIAVPYGAKLLYQELFC   1201
ZM2G133512   MHICQACERVANVIVRAAE--GGGGKKVRGPYCLFCRSAERVVRVDVPYGSKLLYQELFS   1189
Sb07g00460   MHICQACQRVANVIMRPAE---GGSKKVHGPYCMFCRSAERIVRVNVPYGSKLLYQELFC   1239
OsNRPD2b     LHVCRRCQRAAVVVSPAVAADGGGGRKVRGPYCRFCRSAEEVVRVSVPYGAKILYQELFS   1245
Bd_3.1525    MHICQACQRVANVILRS-E----GGKKVHGPYCGFCKSAENILRVNVPYGASLLYKELFC   1286
AtNRPD2a     MHICRKCKTYANVIERTPS----SGRKIRGPYCRVCVSSDHVVRVYVPYGAKLLCQELFS   1160
PtNRPD2      MHICQKCKNVANVIQRGVP----GGRKIRGPYCRVCESVDDLVKVSVPYGAKLLCQELFS   1148
VvNRPD2      MHICRRCKNISNVIQRSVA----GGRKVRGPYCRYCESSEEIVKVNVPYGAKLLCQELFS   1186
OsNRPB2      VHVCEKCGLIAI------------ANLKKNSFECRGCKNKTDIVQVHIPYACKLLFQELMA   1201
AtNRPB2      VHVCEVCGLIAI------------ANLKKNSFECRGCKNKTDIVQVYIPYACKLLFQELMS   1169
```

```
                             1520
NRPD2a       MGICLKFETQVC--------------   1229
Sb01g04210   MGICLKFDTEVC--------------   1228
Sb06g03030   MGICLKFDTEVC--------------   1239
ZM2G128427   MGICLKFETEVC--------------   1129
OsNRPD2a     MGICLRFETEVC--------------   1252
Bd_9.1041    MGICLKFKTEVC--------------   1236
Bd_6.650     MGICLKFQTEIC--------------   1232
Bd_2.4317    MGICLKFQTEIC--------------   1213
ZM2G133512   MGICLKFETEVR--------------   1201
Sb07g00460   MGICLKFDTEIR--------------   1251
OsNRPD2b     MGICLKFDTELI--------------   1257
Bd_3.1525    MGICLKFETEVI--------------   1298
```

```
AtNRPD2a     MGITLNFDTKLC---------------   1172
PtNRPD2      MGISLKFDTRVS---------------   1160
VvNRPD2      MGISLKFETQLC---------------   1198
OsNRPB2      MAIAPRMLTQDNKTGKDQKKR-----   1222
AtNRPB2      MAIAPRMLTKHLKSAKGRQ-------   1188
```
