# Supplementary material for: Diversity of Pol IV Function Is Defined by Mutations at the Maize rmr7 Locus
Source: PLoS Genet. 2009 Nov 20;5(11):e1000706. doi: 10.1371/journal.pgen.1000706 (PMC2775721; doi:10.1371/journal.pgen.1000706)
Supplement: Figure S2 — qRT-PCR analysis. qRT-PCR analysis of the relative abundance of CRM2 LTR RNAs in homozygous nrpd2a-1 mutants relative to heterozygous siblings (error bars are ±1 s.e.m.). (0.17 MB DOC) [file pgen.1000706.s002.doc]

**Figure S2. qRT-PCR analysis**


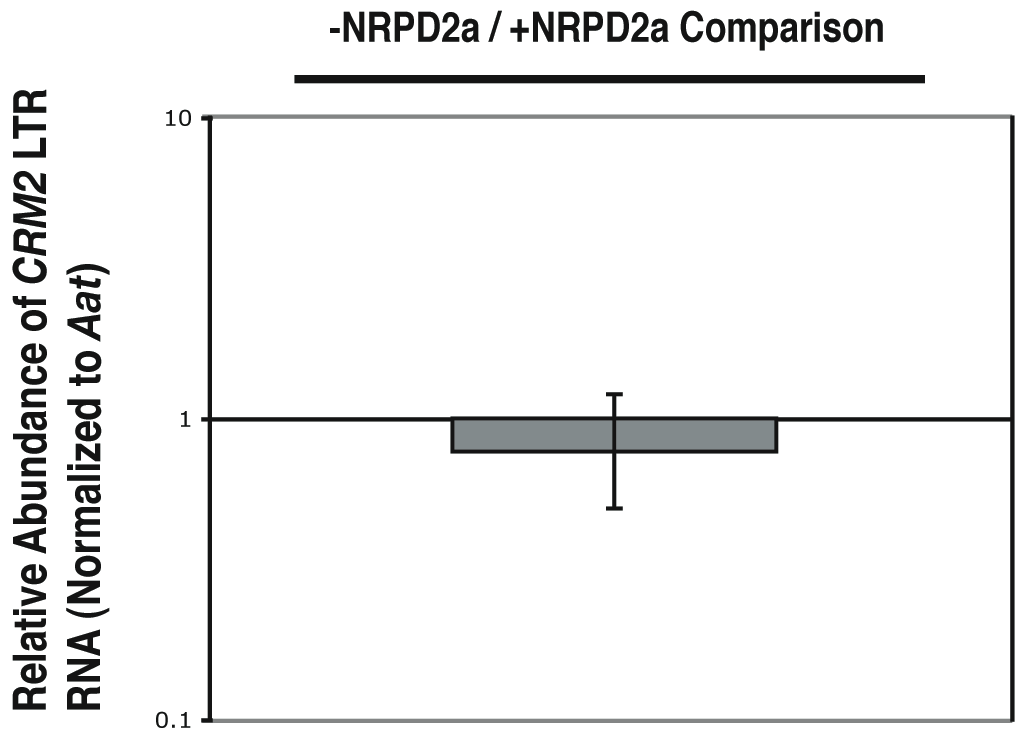


qRT-PCR analysis of the abundance of *CRM2* LTR transcripts in homozygous *nrpd2a-1* mutants relative to heterozygous siblings (error bars are +/- 1 s.e.m.).
