# Supplementary material for: Diversity of Pol IV Function Is Defined by Mutations at the Maize rmr7 Locus
Source: PLoS Genet. 2009 Nov 20;5(11):e1000706. doi: 10.1371/journal.pgen.1000706 (PMC2775721; doi:10.1371/journal.pgen.1000706)
Supplement: Table S1 — ems9750, ems98939, and ems062905 define the rmr7 locus. Progeny anther color scores from crosses between ems9750 (rmr7-1), ems98939 (rmr7-2), and ems062905 (rmr7-3) mutants and plants carrying other rmr and mop mutations. rmr6 is also known as nrpd1. (0.06 MB DOC) [file pgen.1000706.s003.doc]

**Table S1.** ems9750, ems98939, and ems062905 define the *rmr7* locus.

|  | | | | | | | |
| --- | --- | --- | --- | --- | --- | --- | --- |
| Parental genotypes | | | No. of ears | | No. of progeny individuals with specific anther color scores | | |
|  |  |  |  |  |  |  |  |
| Pistillate | | Staminate |  |  | 1-4 | 5-6 | 7 |
|  | | | | | | | |
| *rmr7-1* (ems9750) | | | | | | | |
| + / *mop1-1* | | ems9750 / ems9750 | 1 | | 18 | 0 | 0 |
| + / *mop1-2ems* | | ems9750 / ems9750 | 2 | | 18 | 1 | 0 |
| + / *rmr1-1* | | ems9750 / ems9750 | 1 | | 14 | 0 | 0 |
| + / *rmr1-2* | | ems9750 / ems9750 | 2 | | 21 | 0 | 0 |
| + / *rmr2-1* | | ems9750 / ems9750 | 2 | | 29 | 0 | 0 |
| + / *rmr6-1* | | ems9750 / ems9750 | 2 | | 25 | 0 | 0 |
| *rmr7-2* (ems98939) | | | | | | | |
| + / *mop1-1* | | ems98939 / ems98939 | 2 | | 32 | 0 | 0 |
| + / *mop1-3* | | ems98939 / ems98939 | 2 | | 37 | 0 | 0 |
| *rmr1-1* / *rmr1-1* | | ems98939 / ems98939 | 2 | | 30 | 0 | 0 |
| *rmr2-1* / *rmr2-1* | | ems98939 / ems98939 | 2 | | 36 | 0 | 0 |
| + / *rmr6-1* | | ems98939 / ems98939 | 2 | | 32 | 0 | 0 |
| + / ems9750 | | ems98939 / ems98939 | 2 | | 16 | 16 | 5 |
| *rmr7-3* (ems062905) | | | | | | | |
| + / *mop1-4* | ems062905 / ems062905 | | | 1 | 10 | 0 | 0 |
| + / *rmr1-1* | ems062905 / ems062905 | | | 1 | 13 | 0 | 0 |
| + / *rmr2-1* | ems062905 / ems062905 | | | 1 | 14 | 0 | 0 |
| + / *rmr6-1* | ems062905 / ems062905 | | | 1 | 6 | 0 | 0 |
| + / ems9750 | ems062905 / ems062905 | | | 1 | 15 | 0 | 18 |
|  | | | | | | | |

Progeny anther color scores from crosses between ems9750 (*rmr7-1*), ems98939 (*rmr7-2*), and ems062905 (*rmr7-3*) mutants and plants carrying other *rmr* and *mop* mutations. *rmr6* is also known as *nrpd1*.
