# Supplementary material for: Diversity of Pol IV Function Is Defined by Mutations at the Maize rmr7 Locus
Source: PLoS Genet. 2009 Nov 20;5(11):e1000706. doi: 10.1371/journal.pgen.1000706 (PMC2775721; doi:10.1371/journal.pgen.1000706)
Supplement: Table S2 — rmr7 maps distal to the TB-2Sb breakpoint on 2S. Progeny of independent crosses between Rmr7/rmr7-1 and Pl'/Pl'; TB-2Sb heterozygotes resulted in 15 individuals with dark anthers (ACS scores 5–7). All dark plants showed characteristic 50% pollen abortion and plant phenotypes of 2S monoploids. (0.03 MB DOC) [file pgen.1000706.s004.doc]

**Table S2.** *rmr7* maps distal to the *TB-2Sb* breakpoint on 2S

|  | | | | |
| --- | --- | --- | --- | --- |
| Progeny | Staminate parent | No. of individual progeny with specific anther color scores | | |
|  |  |  | | |
|  |  | 1-4 | 5-6 | 7 |
|  | | | | |
| 10810 | 01-565-3 | 77 | 0 | 5 |
| 11911 | 01-566-11 | 61 | 2 | 8 |
|  | | | | |

Progeny of independent crosses between *Rmr7* / *rmr7-1* and *Pl′* / *Pl′* ; *TB-2Sb* heterozygotes resulted in 15 individuals with dark anthers (ACS scores 5-7). All dark plants showed characteristic 50% pollen abortion and plant phenotypes of *2S* monoploids.
