# Supplementary material for: Diversity of Pol IV Function Is Defined by Mutations at the Maize rmr7 Locus
Source: PLoS Genet. 2009 Nov 20;5(11):e1000706. doi: 10.1371/journal.pgen.1000706 (PMC2775721; doi:10.1371/journal.pgen.1000706)
Supplement: Table S3 — 2S monoploids do not affect paramutation. Progeny anther color scores for crosses between A632 Pl1-Rh/Pl1-Rh and Pl'/Pl'; TB-2Sb heterozygotes. (0.04 MB DOC) [file pgen.1000706.s005.doc]

**Table S3.** 2S monoploids do not affect paramutation

|  | | | | | | |
| --- | --- | --- | --- | --- | --- | --- |
| Progeny | Parents | | No. of individual progeny with specific anther color scores | | | No. of putative *2S* monoploids |
|  |  |  |  |  |  |  |
|  | Pistillate genotype | Staminate individual | 1 | 2 | 3 |  |
|  | | | | | | |
| 41041 | *Pl-Rh* / *Pl-Rh* | 04-850-3 | 1 | 15 | 0 | 4 |
| 41222 | *Pl′* / *Pl′* | 04-850-3 | 5 | 12 | 0 | 3 |
| 41044 | *Pl-Rh* / *Pl-Rh* | 04-851-7 | 5 | 11 | 1 | 3 |
| 41224 | *Pl′* / *Pl′* | 04-851-7 | 15 | 3 | 0 | 3 |
|  | | | | | | |

Progeny anther color scores for crosses between A632 *Pl1-Rh* / *Pl1-Rh* and *Pl*′ / *Pl′*; *TB-2Sb* heterozygotes.
