# Supplementary material for: Diversity of Pol IV Function Is Defined by Mutations at the Maize rmr7 Locus
Source: PLoS Genet. 2009 Nov 20;5(11):e1000706. doi: 10.1371/journal.pgen.1000706 (PMC2775721; doi:10.1371/journal.pgen.1000706)
Supplement: Table S4 — Evaluation of b1 paramutation occurring in individual nrpd2a-1 F1 homozygotes through crosses to homozygous Nrpd2a b1 testers. Two plants, one b1 nrpd2a-1/B-I Nrpd2a; Pl'/Pl' and one B' nrpd2a-1/B' nrpd2a-1; Pl'/Pl', were reciprocally crossed to generate the respective 02–939 (B' nrpd2a-1 homozygote male) and 02–940 (B' nrpd2a-1 homozygote female) progeny sets. Individual F1 progeny plants having dark plant and anther colors were crossed to b1 Nrpd2a/b1 Nrpd2a; Pl-Rh/Pl-Rh testers and the number of testcross progeny plants having a colorless (b1/b1), B'-like (b1/B'), or B-I-like (b1/B-I) phenotype are tallied. (0.05 MB DOC) [file pgen.1000706.s006.doc]

**Table S4.** Evaluation of *b1* paramutation occurring in individual *nrpd2a-1* F_1_ homozygotes through crosses to homozygous *Nrpd2a b1* testers.

|  | | | | | |
| --- | --- | --- | --- | --- | --- |
| Parental *b1* genotype | |  | No. of testcross progeny individuals having a distinct plant phenotype | | |
|  | |  |  | | |
| Maternal | Paternal | F1 individual | colorless | B′ - like | B-I - like |
|  | | | | | |
| *b1 / B-I* | B′ / B′ | 02-939-4 | 10 | 9 | 0 |
| *b1 / B-I* | B′ / B′ | 02-939-11 | 8 | 7 | 0 |
| *b1 / B-I* | B′ / B′ | 02-939-14 | 5 | 6 | 0 |
| B′ / B′ | *b1 / B-I* | 02-940-1 | 8 | 10 | 0 |
| B′ / B′ | *b1 / B-I* | 02-940-10 | 10 | 10 | 0 |
| B′ / B′ | *b1 / B-I* | 02-940-13 | 6 | 10 | 0 |
| B′ / B′ | *b1 / B-I* | 02-940-16 | 10 | 4 | 0 |
| *b1 / B-I* | B′ / B′ | 02-939-3 | 0 | 12 | 5 |
| *b1 / B-I* | B′ / B′ | 02-939-5 | 0 | 11 | 8 |
| *b1 / B-I* | B′ / B′ | 02-939-6 | 0 | 12 | 8 |
| *b1 / B-I* | B′ / B′ | 02-939-12 | 0 | 7 | 9 |
| B′ / B′ | *b1 / B-I* | 02-940-9 | 0 | 13 | 0 |
| B′ / B′ | *b1 / B-I* | 02-940-17 | 0 | 16 | 0 |
|  | | | | | |

Two plants, one *b1 nrpd2a-1 / B-I Nrpd2a ; Pl′ / Pl′* and one *B′ nrpd2a-1 / B′ nrpd2a-1 ; Pl′ / Pl′* , were reciprocally crossed to generate the respective 02-939 (*B'* *nrpd2a-1* homozygote male) and 02-940 (*B'* *nrpd2a-1* homozygote female) progeny sets. Individual F_1_ progeny plants having dark plant and anther colors were crossed to *b1 Nrpd2a / b1 Nrpd2a ; Pl-Rh / Pl-Rh* testers and the number of testcross progeny plants having a colorless (*b1 / b1*), B'-like (*b1 / B'*), or B-I-like (*b1 / B-I*) phenotype are tallied.
