# Supplementary material for: Diversity of Pol IV Function Is Defined by Mutations at the Maize rmr7 Locus
Source: PLoS Genet. 2009 Nov 20;5(11):e1000706. doi: 10.1371/journal.pgen.1000706 (PMC2775721; doi:10.1371/journal.pgen.1000706)
Supplement: Table S5 — Oligonucleotide primers used for nrpd2a sequencing. Oligonucleotide primers designed to amplify the predicted coding regions of the nrpd2a-like gene candidate from genomic DNA sequence by PCR. (0.04 MB DOC) [file pgen.1000706.s007.doc]

**Table S5.** Oligonucleotide primers used for *nrpd2a* sequencing.

| Exon amplified | Forward | Reverse |
| --- | --- | --- |
| 1 | ACAGAAGGACAGTGGGCAAC | GGCTCGGAAGAAGACTTTCC |
| 1 | GATGATAATGGAGCGGGAAA | AGCGAATCAAAAAGCTCCTG |
| 1 | CTATGCCAGCATCACACACC | ATTCACGATGGGGATCTGAG |
| 2 | AGGCAAGCAGCTATTCATCC | CGTCCCCACCTTCACATTTA |
| 3 | CCCAATAGCTTGTTGCGAGT | CAATGAGGTTCGCGTGTTTA |
| 3 | GGTGCGGTGGAAAAATAACT | GGTTGCTTTTAGTTGTTGACAC |
| 3 | CAGTCGTTTGGTTCCATCCT | ATGATTTCGCGAGACCAGAA |
| 4 | AACCTGCACACCAAACATCT | TGACTTAAGACTAAATCCCTACGTACA |
| 5 | GCACATGCAATACTTGGTTGA | CATGTACATTATTTTTCCTTTCATCTT |
| 5 | CAGTGGAAAAGGCACGATTT | CAATGCCAGTTTGGTTGTTG |
| 6 | TGGTGTCATGGCAACTAGGA | CACTGATTATATGTGGGTTATTTTTCA |
| 7 | GGGAAAACCATGGCATACAT | GGATGTGGATATCAAGGGAAAA |

Oligonucleotide primers designed to amplify the predicted coding regions of the *nrpd2a*-like gene candidate from genomic DNA sequence by PCR.
