# Supplementary material for: Diversity of Pol IV Function Is Defined by Mutations at the Maize rmr7 Locus
Source: PLoS Genet. 2009 Nov 20;5(11):e1000706. doi: 10.1371/journal.pgen.1000706 (PMC2775721; doi:10.1371/journal.pgen.1000706)
Supplement: Protocol S1 — Additional materials and methods used for genetic analyses. (0.03 MB DOC) [file pgen.1000706.s008.doc]

**Protocol S1.** Additional materials and methods used for genetic analyses.

Germplasm

Unless otherwise indicated, all stocks are homozygous for genes encoding functional enzymes of the anthocyanin biosynthetic pathway and each contains either *R-r*-like haplotypes conferring kernel aleurone color in combination with dominant *colored aleurone1* (*c1*) alleles or *r-r*-like haplotypes (non-functional for seed color only). Specific coding regions of both *R-r* and *r-r* haplotypes are expressed in somatic tissues and confer pigment to seedling sheaths and anthers in combination with expression of functional PL1 protein. Three related *Pl-Rh / Pl-Rh* converted inbred lines (W23, A619 and A632) have been previously described [1]. The *Pl1-Rhoades* allele in each line spontaneously changes to *Pl′* at distinct frequencies (A619, ~0.02%; W23, ~0.07%; A632, ~1-10%).

The *TB-2Sb* interchange [2] obtained from the Maize Genetics Cooperation Stock Center (USDA-ARS, Urbana, IL) was maintained in heterozygous condition. Plants heterozygous for the *TB-2Sb* interchange display ~25% pollen abortion and can produce segmental monoploids via non-disjunction of *B* centromeres in the cell division giving rise to the sperm cells [2]. The *TB-2Sb* genotype was confirmed in every generation by visually examining freshly shed pollen and by the occurrence of kernels with pale yellow endosperms on testcross ears of +/*albescent plant* (*al1*) plants. With the resident *Y1* alleles, endosperms of *al1*/*al1*/+ and *al1*/*al1*/+/+ genotype are yellow, while endosperms of *al1*/*al1*/- genotype are pale yellow [3]. To synthesize the *Pl′* / *Pl′* line, a *TB-2Sb* heterozygote was first crossed by K55 *Pl1-Rhoades* (*Pl′* / *Pl′*) and then *TB-2Sb* heterozygous progeny were crossed by K55 *Pl1-Rhoades* (*Pl-Rh* / *Pl-Rh*). The K55 backcross produced equal numbers of progeny with fully pigmented anthers (*pl1-coop* / *Pl-Rh*) and lightly pigmented anthers (*Pl*′ / *Pl-Rh*). The desired *TB-2Sb* stock was maintained by repeated backcrosses with the K55 *Pl-Rh* / *Pl-Rh* line. All other *Pl1-Rhoades* lines and other stocks have been previously described [4,5,6]. Descriptions of the EMS-derived mutagenesis materials have been previously reported [7,8]. The *T6-9 (043-1)* interchange (referred to here as *T Pl*′) was used to identify chromosomes carrying *Pl1-Rhoades* of *Pl*′ state and has been previously described [1].

Analysis of new *rmr*-type mutations

The ems9750 mutation was first recognized in sand bench screening of an ems-derived M_2_ progeny (9750) as 3/23 seedlings had a dark pigment phenotype similar to that of *Pl-Rh / Pl-Rh* individuals. All three mature plants grown from these darkly colored seedlings were small and had Pl-Rh-like anthers. The ems98939 mutation was similarly recognized as 2/29 seedlings were darkly pigmented. One hundred additional individuals from this M_2_ progeny were grown as seedlings and then 10 of the darkest and 10 of the lightest were transplanted into the field where mature flowers were evaluated for pigmentation. The ten plants derived from the lightest colored seedlings had only Pl'-like anthers, while the 9 surviving plants derived from the darkest colored seedlings had exclusively Pl-Rh type anthers. One other *rmr*-type mutation was directly recognized in the ems-derived M_2_ progeny, 062905, via the appearance of 3/37 plants having Pl-Rh-like anthers.

Transmission frequencies of the mutations were assessed by evaluating anther phenotypes of F_2_ progeny derived from self-pollinations of F_1_ individuals mentioned in the Results section. Pl-Rh-like phenotypes were recovered in 34/154 (22%) F_2_ plants derived from seven independent self-pollinations of F_1_ plants (9750-derived). The observed frequency of mutant phenotypes (22%) is not significantly different from the 25% expected from a single locus recessive mutation (χ^2^ = 0.53; *P* > 0.05). Pl-Rh-like phenotypes were also recovered in 4/16 (25%) F_2_ plants derived from *wx/wx/wx* mutant kernels selected from three independent self-pollinations of F_1_ plants (98939-derived). The observed frequency of mutant phenotypes is exactly as expected from a single locus recessive mutation. Given that the mutant *waxy* allele is linked to a *T6-9* breakpoint and *pl1*, this result indicates that the ems98939 mutation is genetically unlinked to the *pl1* locus.

Genetic tests (Table S1) indicated that all three ems-derived mutations fail to genetically complement each other with regard to maintaining the repressed *Pl'* state suggesting that they define mutant alleles of the same locus. Similar tests with mutations defining the *mop1* [9], *rmr1* [8], *rmr2* [7], and *rmr6 / nrpd1* [1] loci showed that all the new mutations genetically complement these previously identified mutations (Table S1).

Quantitative RT-PCR analysis and transcript abundance calculations

Quantitative RT-PCR was carried out on random-primed cDNA generated as described above. All reactions were performed on an ABI 7300 real-time cycler (Applied Biosystems) using the DyNAmo HS SYBR Green qPCR kit (New England Biolabs) according to the manufacturer’s instructions. Averages of mutant and non-mutant *CRM2* LTR expression levels were derived from data from five technical replicates of two different biological replicates of each genotype (*+ / nrpd2a-1* and *­nrpd2a-1 / nrpd2a-1*) using the 2^-ΔΔC^_T_ method [11]. Mutant and non-mutant *CRM2* transcript levels were normalized to *Aat* transcript levels; this calculation assumes equal efficiencies of amplification for the internal control *Aat* products and the experimental *CRM2* products. Error bars shown in Figure S2 represent 1 standard error of the mean (s.e.m.) estimated for replicate mutant and non-mutant *Aat* and *CRM2* C_T_ values and carried through to the final calculation of the 2^-ΔΔC^_T_ value by standard propagation of error methods [11].

### Literature Cited

1. Hollick JB, Kermicle JL, Parkinson SE (2005) *Rmr6* maintains meiotic inheritance of paramutant states in *Zea mays*. Genetics 171: 725—740.

2. Beckett JB (1978) *B-A* translocations in maize. I. Use in locating genes to chromosome arms. J Hered 69: 27—36.

3. Phipps IF (1929) Relations of virescent seedlings in maize. Cornell Univ. Agric. Exp. Stn Memoir 125:1—63.

4. Hollick JB, Patterson GI, Coe EH Jr, Cone KC, Chandler VL (1995) Allelic interactions heritably alter the activity of a metastable maize *pl* allele. Genetics 141: 709—719.

5. Hollick JB, Chandler VL (1998) Epigenetic allelic states of a maize transcriptional regulatory locus exhibit overdominant gene action. Genetics 150: 891—897.

6. Gross SM, Hollick JB (2007) Multiple *trans*-sensing interactions affect meiotically heritable epigenetic states at the maize *pl1* locus. Genetics 176: 829—839.

7. Hollick JB, Chandler VL (2001) Genetic factors required to maintain repression of a paramutagenic maize *pl1* allele. Genetics 157: 369—378.

8. Hale CJ, Stonaker JL, Gross SM, Hollick JB (2007) A novel Snf2 protein maintains *trans*-generational regulatory states established by paramutation in maize. PLoS Biol 5(10): e275. doi:10.1371/journal.pbio.0050275

9. Dorweiler JE, Carey CC, Kubo KM, Hollick JB, Kermicle JL, et al. (2000) *mediator of paramutation1 (mop1)* is required for the establishment and maintenance of paramutation at multiple maize loci. Plant Cell 12: 2101—2118.

10. Mroczek RJ, Dawe RK (2003) Distribution of retroelements in centromeres and neocentromeres of maize. Genetics 165: 809—819.

11. Livak KJ, Schmittgen.TD (2001) Analysis of relative gene expression data using real-time quantitative PCR and the 2(-Delta Delta C(T)) Method. Methods 25: 402—40
